# Mammalian splicing factor SF1 interacts with SURP domains of U2 snRNP-associated proteins

**DOI:** 10.1093/nar/gkv952

**Published:** 2015-09-29

**Authors:** Angela Crisci, Flore Raleff, Ivona Bagdiul, Monika Raabe, Henning Urlaub, Jean-Christophe Rain, Angela Krämer

**Affiliations:** 1 Department of Cell Biology, Faculty of Sciences, University of Geneva, CH-1211 Geneva 4, Switzerland; 2 Bioanalytical Mass Spectrometry, Max Planck Institute for Biophysical Chemistry, D-37077 Göttingen, Germany; 3 Bioanalytics, Institute for Clinical Chemistry, University Medical Center Göttingen, D-37075 Göttingen, Germany; 4 Hybrigenics Services, F-75014 Paris, France

## Abstract

Splicing factor 1 (SF1) recognizes the branch point sequence (BPS) at the 3′ splice site during the formation of early complex E, thereby pre-bulging the BPS adenosine, thought to facilitate subsequent base-pairing of the U2 snRNA with the BPS. The 65-kDa subunit of U2 snRNP auxiliary factor (U2AF65) interacts with SF1 and was shown to recruit the U2 snRNP to the spliceosome. Co-immunoprecipitation experiments of SF1-interacting proteins from HeLa cell extracts shown here are consistent with the presence of SF1 in early splicing complexes. Surprisingly almost all U2 snRNP proteins were found associated with SF1. Yeast two-hybrid screens identified two SURP domain-containing U2 snRNP proteins as partners of SF1. A short, evolutionarily conserved region of SF1 interacts with the SURP domains, stressing their role in protein–protein interactions. A reduction of A complex formation in SF1-depleted extracts could be rescued with recombinant SF1 containing the SURP-interaction domain, but only partial rescue was observed with SF1 lacking this sequence. Thus, SF1 can initially recruit the U2 snRNP to the spliceosome during E complex formation, whereas U2AF65 may stabilize the association of the U2 snRNP with the spliceosome at later times. In addition, these findings may have implications for alternative splicing decisions.

## INTRODUCTION

Pre-mRNA splicing is essential for eukaryotic gene expression and one of the most versatile mechanisms to increase proteome diversity through alternative splice site choices (1). The reaction is catalyzed by the spliceosome, a multi-megadalton complex (2). Five small nuclear ribonucleoprotein particles (U1, U2, U4, U5 and U6 snRNPs) and more than 100 non-snRNP proteins assemble in a step-wise fashion on the pre-mRNA through networks of RNA–RNA, RNA–protein and protein–protein interactions to form the catalytic center of the spliceosome for intron removal.

Essential sequence elements at the 5′ and 3′ splice sites are recognized at the onset of spliceosome assembly (2). U1 snRNP binds to the 5′ splice site, whereas SF1 and the U2 snRNP auxiliary factor (U2AF) recognize distinct sequences at the 3′ splice site. SF1 and the large subunit of U2AF (U2AF65) interact with one another and cooperatively bind the branch point sequence (BPS) and the adjacent polypyrimidine tract, respectively (3–8). The small U2AF subunit (U2AF35) recognizes the 3′ splice site AG dinucleotide (9). Together these interactions assemble the early complex E, which is converted into pre-splicing complex A by incorporation of the U2 snRNP. This is accomplished through interaction of the U2 snRNP-associated SF3b155 with U2AF65 (10), binding of U2 snRNP proteins to and adjacent to the BPS (11–13) and base pairing of the U2 snRNA with the BPS. These events are thought to displace SF1 from the spliceosome (14). In addition, displacement of the *Saccharomyces cerevisiae* ortholog of SF1, termed branch point-binding protein (BBP; also known as MSL5 or YLR116W) is thought to require the DExD-box helicase Sub2p (15–17). The following steps of spliceosome assembly involve binding of the remaining snRNPs and additional non-snRNP proteins, juxtaposition of the splice sites and dynamic remodeling of the complexes leading to the formation of the catalytic center, followed by intron removal in two catalytic steps (2).

SF1 was initially identified as a protein required for pre-spliceosome assembly (18,19). It is evolutionarily conserved and yeast BBP was shown to function in the formation of the early commitment complex CC2, which together with CC1 is the equivalent of the human E complex (14). SF1 is essential in *S. cerevisiae*, *Caenorhabditis elegans* and mammalian cells (4,20–23), but surprisingly, initial RNA interference-mediated knockdown of SF1 in human cells did not affect the splicing of several pre-mRNAs tested (22). Similarly, biochemical or genetic depletion of SF1/BBP from human extracts or yeast cells only marginally affected splicing activity, possibly due to a kinetic role for SF1 in spliceosome assembly (14,24). Later experiments demonstrated that SF1 is not involved in the splicing of all introns, but influences alternative splicing decisions (25–28). Thus, altered or mis-splicing of essential genes in the absence of SF1 could reflect its requirement for viability. In addition to its role in splicing, SF1 has been implicated in nuclear pre-mRNA retention and transcriptional repression (29,30), functions that may contribute to or be the cause of SF1's essential phenotype.

To better understand SF1 function in splicing and potentially other processes, we set out to identify interacting proteins by co-immunoprecipitation (co-IPs) from HeLa cell nuclear extracts combined with mass spectrometry (MS) and in yeast two-hybrid (Y2H) screens with human SF1. Our results confirm that the major role for SF1 is in early stages of the splicing reaction. We demonstrate a novel function of SF1, which is essential for efficient spliceosome assembly, in the initial recruitment of the U2 snRNP through direct interactions with two U2 snRNP-associated proteins. The isolation of Y2H partners moreover suggests interactions of SF1 with additional proteins implicated in splicing, but also in other processes and identifies domains potentially mediating these interactions.

## MATERIALS AND METHODS

### Co-IP of SF1-interacting proteins and MS

HeLa cell nuclear extract (corresponding to 2 mg of total protein) was incubated in the absence or presence of 0.3 mg/ml RNase A (Sigma) for 15 min at room temperature followed by pre-clearing for 1 h at 4°C with Protein G Sepharose (GE Healthcare) equilibrated in IP buffer (50 mM Tris-HCl, pH 7.9, 100 mM NaCl, 0.05% Nonidet P-40 [NP-40] and 0.5 mM dithiothreitol [DTT]). Unbound material was incubated for 1 h at 4°C with Protein G Sepharose-coupled anti-SF1 (Abnova; H7536-MO1A) or control mouse IgG (Sigma, P4810), followed by centrifugation at 2000 rpm in a microfuge for 2 min at 4°C. Unbound material was saved and the beads were washed three times with 1 ml IP buffer. Bound material was eluted with NuPAGE LDS sample buffer (Life Technologies) and separated in NuPAGE Novex 4–12% Bis-Tris protein gels (Life Technologies), followed by Coomassie blue staining. Proteins were in-gel trypsin-digested and extracted according to Shevchenko et al. (31). Peptides were analyzed in an LTQ Orbitrap XL mass spectrometer (Thermo Fisher Scientific) under standard conditions. Proteins were identified by searching fragment spectra against the NCBI non-redundant database using Mascot as search engine. The number of product ion spectra (total spectrum count) of identified peptides was compared between anti-SF1 and control-IgG samples. Proteins with more than 15% of spectra in control IgG versus anti-SF1 were eliminated. Proteins with five or more spectrum counts are shown in Table 1; those with less than five spectrum counts, in addition to proteins assumed to represent common contaminants, are listed in Supplementary Table S1.

**Table 1.** Proteins immunoprecipitated with anti-SF1 antibodies from HeLa cell nuclear extracts

				SF1		IgG	
Name^1^	Symbol^2^	Gene ID^3^	Group^4^	− RNase	+ RNase	− RNase	+ RNase
Sm-B/B’	SNRPB	6628	Sm	51	20	1	2
Sm-E	SNRPE	6635	Sm	48	15	1	2
Sm-G	SNRPG	6637	Sm	7	13	1	1
U1–70K	SNRNP70	6625	U1	15	10	0	0
U1-A	SNRPA	6626	U1	39	29	1	2
SRPK1	SRPK1	6732	U1	0	6	0	0
U2-A’	SNRPA1	6627	U2	27	19	0	1
U2-B”	SNRPB2	6629	U2	12	10	0	0
SF3a120*	SF3A1	10291	U2	41	47	6	5
SF3a60	SF3A3	10946	U2	23	28	1	2
SF3b155	SF3B1	23451	U2	76	66	9	5
SF3b145	SF3B2	10992	U2	47	50	0	3
SF3b130	SF3B3	23450	U2	96	93	6	7
SF3b14a	SF3B14	51639	U2	17	10	1	1
SF3b10	SF3B5	83443	U2	7	9	0	0
CHERP*	CHERP	10523	U2-related	34	15	0	0
SF3b125	DDX42	11325	U2-related	4	19	0	0
hPRP43	DHX15	1665	U2-related	55	66	6	10
SPF31	DNAJC8	22826	U2-related	7	10	1	1
**PUF60**	PUF60	22827	U2-related	22	26	0	1
**SPF45**	RBM17	84991	U2-related	10	7	0	0
U2SURP*	SR140	23350	U2-related	41	41	1	0
**U2AF65**	U2AF2	11338	U2-related	27	34	0	1
U2AF35	U2AF1	7307	U2-related	10	11	1	1
**FBP11**	PRPF40A	55660	A	8	15	0	0
S164	RBM25	58517	A	16	14	0	0
RBM39	RBM39	9584	A	0	8	0	0
SF1	SF1	7536	A	185	193	0	0
SUGP1*	SF4	57794	A	0	6	0	0
SRp40	SFRS5	6430	SR	8	4	0	0
SRp55	SFRS6	6431	SR	8	4	0	0
9G8	SFRS7	6432	SR	22	18	3	1
CYPH	PPIH	10465	U4/U6	7	5	1	0
hPRP31	PRPF31	26121	U4/U6	6	7	0	0
hPRP28	DDX23	9416	U5	1	5	0	0
hPRP6	PRPF6	24148	U5	18	9	1	2
hSNU66	SART1	9092	U4/U6.U5	7	3	0	0
CDC5L	CDC5L	988	Prp19	5	7	0	0
hSmu1	SMU1	55234	B	1	7	0	0
SRm300	SRRM2	23524	B (act)	0	7	0	0
TOE1	TOE1	114034	C2	2	5	0	0
Acinus	ACIN1	22985	EJC/TREX	18	26	0	0
THOC2	THOC2	57187	EJC/TREX	9	4	0	0
ARS2B	ARS2	51593	mRNA	52	45	2	2
CBP80	NCBP1	4686	mRNA	24	24	1	3
CBP20	NCBP2	22916	mRNA	9	0	1	0
ZC3H18	ZC3H18	124245	mRNA	9	11	0	0
ZNF207	ZNF207	7756	misc.	5	2	0	0
	ANXA6	309		5	0	0	0
	BRD2	6046		0	18	0	0
	CDK11B	984		3	5	0	0
	CKB	1152		5	3	0	0
	EIF3A	8661		12	3	0	0
	EIF3C	8663		6	1	0	0
	HSPA4L	22824		13	16	1	2
	PCNP	57092		8	2	0	0
	SAFB2	9667		7	1	0	0
	ZFR	51663		10	0	0	0
	ZNF598	90850		0	5	0	0

Proteins were separated by 1D PAGE (see Supplementary Figure S1) and identified by LC-MSMS. The total spectrum count is shown. The experimental set-up is described in the Materials and Methods section. SF1 or IgG, proteins precipitated by anti-SF1 antibodies or control IgG coupled to Protein G Sepharose; - RNase or + RNase, HeLa extracts were incubated without (−) or with (+) RNase A prior to IP. Raw data were filtered (see Materials and Methods section) and the final list was compared to 244 spliceosomal proteins annotated in Supplementary Table S1 of Hegele et al. (49).

^1^Protein name commonly used in the splicing field. Proteins shown in bold are known SF1 partners; underlined, proteins also found in Y2H screens (Table 3); *, proteins with SURP domains.

^2^NCBI ENTREZ symbol.

^3^NCBI ENTREZ GeneID.

^4^Operational classification of spliceosomal proteins according to Hegele et al. (49).

### Y2H analysis

Y2H screens were performed with the partial coding sequence of human SF1 (amino acids 1–441; GenBank accession number gi: 295842308), which was PCR-amplified and cloned into pB27 as a C-terminal fusion to LexA (N-LexA-SF1-C). The construct was verified by sequencing and used as a bait to screen random-primed and dT-primed cDNA libraries constructed in pP6 (Table 2) with a mean insert size of 900 bp. Library construction has been described (32). pB27 and pP6 are derived from pBTM116 and pGADGH, respectively (33,34).

**Table 2.** Libraries used in Y2H screens

Library name	Description	Number of clones tested	Histidine- positive
CEMC7_RP^1^	Human T cell line CEMC7	53 × 10^6^	70
HBMEC_RP^1^	Human bone marrow endothelial cells (cloned cells transformed by SV40)	58 × 10^6^	353
HTH_RP^1^	Human thymocytes, CD4+CD8+ double positive cells from children	35 × 10^6^	104
HTH_dT^2, 3^	Human thymocytes, CD4+CD8+ double positive cells from children	88 × 10^6^	97
PLA_RP^1^	Human placenta	105 × 10^6^	211
MANE_RP^1^	Mouse adult neurosphere cells (free-floating clusters of neural stem cells)	65 × 10^6^	284
MKI_RP^1^	Total kidney, adult C57BL/J6 mice, age 8–10 weeks	102 × 10^6^	144
MPC_RP^1^	Mouse pancreatic cells (beta-cell line, betaTC-tet)	71 × 10^6^	143

^1^RP, random-primed.

^2^dT, oligo-dT-primed.

^3^Performed in the presence of 0.5 mM 3-amino-1,2,4-triazole.

Baits were screened using a mating approach with YHGX13 (Y187 ade2–101::loxP-kanMX-loxP, matα) and L40ΔGal4 (mata) yeast strains as described (35). The number of clones tested and histidine-positive clones are listed in Table 2. Prey fragments of positive clones were PCR-amplified and sequenced at the 5′ and 3′ junctions. Preys from the dT-primed library were only sequenced at the 5′ junctions. The resulting sequences were used to identify the corresponding interacting proteins in the GenBank database (http://www.ncbi.nlm.nih.gov/gene/) with a fully automated procedure. A confidence score (Predicted Biological Score, PBS) was attributed to each interaction as described (32).

### Data set

The protein interactions from this publication have been submitted to the IntAct Molecular Interaction Database (36) at the International Molecular Exchange Consortium (IMEx; http://www.imexconsortium.org/) and assigned the identifier IM-23671. The data are presented in Supplementary Tables S2–9 and Supplementary Figures S4–S11 and can also be browsed with the PIMRider data analysis software at https://pimr.hybrigenics.com/ (SF1 project).

### Cloning procedures

DNAs were amplified with the Expand High Fidelity PCR System (Roche) according to the manufacturer's instructions and suitable primers. Correct cloning was verified by sequencing (Microsynth, Switzerland).

DNAs for bacterial expression of the GST-tagged SURP1 of SF3a120 (amino acids 38–113), SURP2 of SFSWAP (amino acids 197–272) and the single SURP domain of CHERP (amino acids 1–75) were cloned into the Gateway vector pDEST15 (Life Technologies). The plasmid encoding the GST-tagged U2AF homology motif (UHM) of U2AF65 (amino acids 367–475) has been described (4).

Plasmids for bacterial expression of SF1 proteins were cloned as follows: C-terminal truncations carrying N-terminal His_6_-tags were cloned into the BamHI and EcoRI sites of pTrcHisA (Life Technologies). N-terminal deletions were cloned into the NcoI and EcoRI sites of pETMBP-1a (a gift of Michael Sattler, Helmholtz Center and Technical University, Munich), encoding proteins with N-terminal His_6_-MBP tags. Internal deletions of SF1-C370 in pETMBP-1a were generated by replacing the deleted sequences with one (Δ306–326 and ΔZn) or two (ΔKH) KpnI sites, encoding Gly and Thr. Internal sequences of SF1 (127–302, 295–335 and 304–326) were cloned into the NcoI/EcoRI sites of pETMBP-1a.

For transient expression of N-terminal GFP fusion proteins in HeLa cells, SF1 sequences were cloned into the Gateway vector pDEST53 (Life Technologies).

Templates for *in vitro* transcription of 3′ splice site pre-mRNAs were generated by HindIII and BstEII digestion of a pBluescript plasmid encoding the region spanning exons 1 and 2 of AdML pre-mRNA (37). The 3′ overhangs were blunt-ended with T4 DNA polymerase (Promega) followed by religation. The resulting pre-mRNA contains 51 nts of vector sequences, 75 nts of the 3′ end of AdML intron 1 and 38 nts of exon 2. This plasmid was used to mutate the original AdML BPS (UACUUAU) to a consensus (UACUAAC) or weak (AAUUCAC) BPS with the GeneTailor Site-Directed Mutagenesis System (Life Technologies).

### Bacterial expression and purification of recombinant proteins

Plasmids encoding GST-tagged SURP domains and His_6_-MBP-tagged SF1 proteins were transformed into *E. coli* strain BL21 by heat shock. Plasmids encoding GST, GST-U2AF65-UHM and His_6_-tagged SF1 proteins were transformed into *E. coli* strain XL1-Blue by heat shock. Proteins were expressed for 4 h at 37°C after addition of isopropyl-β-D-thiogalactopyranoside (IPTG) to a final concentration of 1 mM. Cells were harvested by centrifugation at 5000 × g for 10 min.

Cells expressing GST-tagged proteins were lysed in PBS supplemented with complete protease inhibitors (Roche) by sonication on ice and supplemented with Triton X-100 to a final concentration of 1%. Proteins were purified with 500 μl of a 50% suspension of glutathione agarose (Sigma) equilibrated in PBS. Unbound proteins were removed by washing three times with 10 ml PBS and GST-tagged proteins were eluted with 5 mM glutathione and 50 mM Tris-HCl, pH 8.0.

Cells expressing His_6_- or His_6_-MBP-tagged proteins were lysed in 10 mM Tris-HCl, pH 8.0, 100 mM NaCl, 50 mM Na phosphate, 8 M urea and complete protease inhibitors. Proteins were purified on TALON Metal Affinity Resin (BD Biosciences). Unbound material was removed by three washes with lysis buffer containing 0.4 M NaCl and 20 mM imidazole and bound proteins were eluted with 0.1 M EDTA, pH 8.0.

All recombinant proteins were dialyzed against D buffer (38) supplemented with 3 mM MgCl_2_ and stored at −80°C. Purified proteins were quantified by SDS-PAGE and Coomassie blue staining.

### GST pull-down assays

Reactions containing 20 μl of packed glutathione-agarose beads (Sigma) and 0.056 nmole (corresponding to ≈2 μg) of GST fusion proteins in a total volume of 200 μl NETN (20 mM Tris-HCl, pH 8.0, 50 mM NaCl, 0.5% NP-40, 0.5 mM EDTA) were incubated for 30 min at 4°C and washed twice with 500 μl NETN. His_6_- or His_6_-MBP tagged proteins were added in a two-fold molar excess and the reaction mixture was incubated for 45 min at 4°C in a total volume of 200 μl NETN. Where indicated, purified proteins were treated with 0.3 mg/ml of RNase A for 20 min at room temperature and centrifuged at maximal speed in a microfuge for 5 min prior to GST pull-down. Unbound proteins were removed by three NETN washes. Proteins were eluted from the beads with SDS sample buffer for 5 min at 95°C and separated by SDS-PAGE followed by western blotting (39). Input and bound proteins were detected with mouse monoclonal anti-His_6_ (Sigma; H1029); the amount of GST-tagged proteins was verified by incubation with rabbit anti-GST (A.K., unpublished). Goat anti-mouse and anti-rabbit IRDye800 and 680LT (LI-COR) were used as secondary antibodies and immune complexes were visualized with the Odyssey Fc imaging system (LI-COR Biosciences).

### Cell culture, transient transfection and preparation of HeLa cell lysates and nuclear extract

HeLa cells were grown at 37°C with 5% CO_2_ in DMEM (Sigma) supplemented with 10% FBS (Sigma), 2 mM L-glutamine (Gibco), 100 u/ml penicillin and 100 μg/ml streptomycin (Gibco). Cells were plated in 10-cm culture dishes 24 h prior to transfection. Cells were transfected at 60–70% confluency with polyethylenimine (Brunschwig) according to the manufacturer's instructions and collected 72 h later. Whole cell lysates were prepared by lysis of transfected cells in 50 mM Tris-HCl, pH 7.5, 100 mM NaCl, 1% NP-40, 1 mM DTT, 2 mM EDTA and complete protease inhibitors for 30 min at 4°C followed by centrifugation at 16 000 x g for 5 min at 4°C. HeLa cell nuclear extracts were prepared according to Dignam et al. (38) and dialyzed against buffer D.

### Co-IPs

HeLa cell nuclear extract was incubated with 0.3 mg/ml RNase A (Sigma) for 20 min at room temperature and centrifuged in a microfuge at maximal speed for 5 min. The supernatant (corresponding to 100 μg of total protein) was incubated for 1 h at 4°C with Dynabeads Protein G (Invitrogen) coated with anti-SF1 or control mouse IgG. The unbound fraction was kept for western blotting and beads were washed four times with 1 ml of 50 mM Tris-HCl, pH 7.5, 100 mM NaCl, 0.05% NP-40 and 0.5 mM DTT. Bound proteins were eluted with SDS sample buffer for 5 min at 95°C. Input, unbound and bound material were separated by SDS-PAGE followed by western blotting with the following antibodies: mouse monoclonal anti-CHERP (Santa-Cruz; Sc-100650), rabbit anti-H1 (Santa-Cruz; Sc-10806), mouse monoclonal anti-SF1 (Abnova; H7536-MO1A), rabbit anti-SF3a60 (40), mouse monoclonal anti-SF3a66 (41), rabbit anti-SF3a120 (42), rabbit anti-SFSWAP (Aviva; ARP 40524) and mouse monoclonal anti-U2AF65 (Sigma; U4758). Secondary antibodies and detection of immune complexes were as above.

For precipitation of transiently expressed GFP-tagged SF1 and associated proteins, HeLa whole cell lysate (corresponding to 6 mg of total protein per reaction) was treated with RNase A as above and incubated for 1 h at 4°C with Dynabeads Protein G-coupled goat anti-GFP (a gift of Karla Neugebauer, Yale University). Washing, elution, SDS-PAGE and western blotting were performed as above. Proteins were detected with anti-SF3a120, anti-U2AF65 and rabbit anti-GFP (Invitrogen; A-11122).

### Immunodepletion

SF1 was immunodepleted from HeLa cell nuclear extract adjusted to 600 mM KCl by three passages over Dynabeads Protein G-coupled anti-SF1. The depleted extract was dialyzed against buffer D (38) and stored at −80°C. Mock depletions were performed as above in the absence of antibody.

### Spliceosome assembly

AdML pre-mRNAs with a consensus or weak BPS were synthesized with T3 RNA polymerase (Promega) in the presence of [α-^32^P]UTP and gel-purified (37,43). Biological triplicates of spliceosome assembly were performed in 10-μl reactions in the presence of 10% untreated, mock- or SF1-depleted HeLa nuclear extract, 10 mM Hepes-KOH, pH 7.9, 50 mM KCl, 0.05 mM EDTA, 0.25 mM DTT, 1 μg tRNA and 1.6 pmol pre-mRNA at 30°C for the times shown in the figure legends. Where indicated, reactions were complemented with 0.022, 0.22 or 2.2 pmol SF1-C370 or SF1–302. Reaction products were separated in native 4% polyacrylamide gels (44). Gels were dried and exposed to PhosphorImager screens. Quantification was done with the Molecular Imager FX (BioRad) and software Quantity One V 4.2.1 (BioRad).

### UV cross-linking and IP of U2AF65

Spliceosome assembly was performed as above in 20-μl reactions in the presence of 25% mock- or SF1 depleted extract and 18 pmol pre-mRNA. Samples were incubated at 30°C for 15 min, UV cross-linked and treated with RNase A as described (45). Dynabeads Protein G coupled with control IgG or anti-U2AF65 and 100 μl buffer D (38) were added and samples were incubated for 1.5 h at 4°C with rotation. After centrifugation, the beads were washed four times with 600 μl of 50 mM Tris-HCl, pH 7.5, 0.5 M NaCl, 1% NP-40 and 0.5 mM DTT, and once with 50 mM Tris-HCl, pH 7.5 and 1% NP-40. Bound material was eluted by boiling in SDS loading buffer for 5 min, followed by centrifugation and separation by 10% SDS-PAGE. Gels were dried, exposed to a PhosphorImager screen and signals were quantified as above.

## RESULTS

### The U2 snRNP co-immunoprecipitates with SF1

To identify potential novel partners of SF1, we performed co-IPs of HeLa cell nuclear extracts followed by MS. Extracts were incubated in the absence or presence of RNase A, pre-cleared on Protein G Sepharose and aliquots were incubated with Protein G Sepharose-coupled control mouse IgG or a monoclonal antibody directed against the N-terminal 110 amino acids of SF1. Eluted proteins were separated by 1D PAGE (Supplementary Figure S1), in-gel trypsin-digested and analyzed by liquid chromatography tandem mass spectrometry (LC-MSMS).

A total of 58 proteins were specifically precipitated by anti-SF1 (Table 1), four of which were previously reported to directly interact with SF1: FBP11/PRPF40A, PUF60, SPF45/RBM17 and U2AF65/U2AF2, (3,4,20,46–48). Forty-seven proteins are represented in a list of 244 annotated spliceosomal proteins (see 49). Among these, 29 are associated with the U1 or U2 snRNPs or function in pre-splicing complex A assembly. Eight are part of the U4/U6, U5 or U4/U6.U5 snRNPs or function after A complex formation. Three members of the SR family of proteins with functions early and late during spliceosome assembly were found, in addition to six proteins with roles in mRNA export or mRNA binding and one miscellaneous protein associated with the spliceosome. Thus, consistent with the function of SF1 at the initial steps of spliceosome assembly, more than half of the proteins co-precipitating with SF1 are components of early spliceosomes, compared to 52 of the annotated early spliceosomal proteins (excluding SR and hnRNP proteins), whereas only a small percentage (eight of 103 proteins) are associated with later complexes. An additional 14 spliceosome-associated proteins were detected below the cut-off used for the data in Table 1 and are listed in Supplementary Table S1, together with proteins not known to be part of the spliceosome or to be common contaminants.

A closer inspection of the early spliceosomal proteins precipitated by anti-SF1 revealed the presence of nine of the 12 U2 snRNP-specific proteins, as well as nine of 11 proteins more loosely associated with the U2 snRNP (Table 1, U2 and U2-related, respectively; see 50). This finding was surprising, since the U2 snRNP is thought to replace SF1 from the spliceosome during the complex E to A transition by binding of the U2 snRNP-associated SF3b155 to the U2AF65-UHM, a site occupied by SF1 in complex E (14,51–53) and base pairing of the U2 snRNA with the BPS. Moreover, SF1 has not been found among U2 snRNP-associated proteins (50). However, although the U2 snRNA base-pairs with the BPS only in complex A (54), the U2 snRNP has been detected already in complex E (55–57).

Eleven proteins isolated by co-IP are not known to be related to splicing (Table 1). Most are nuclear proteins with functions in RNA binding and/or transcription. The presence of some of these proteins may be due to interactions with other components of the splicing machinery that are directly or indirectly associated with SF1.

### SF1 interacts with U2 snRNP-associated proteins in the Y2H system

Information regarding potential direct SF1 partners was obtained from Y2H screens with residues 1–441 of human SF1 fused to the C terminus of the LexA DNA binding domain. This portion of SF1 contains all domains required for function in *in vitro* splicing assays (4), but lacks most of the Pro-rich C-terminal region, previously shown to strongly activate transcription in the Y2H system (4). cDNAs of four human and three mouse tissues or cell lines fused to the Gal4 activation domain were tested for interactions (Table 2).

As summarized in Table 3 (and listed in detail in Supplementary Tables S2–S9), previously described SF1 partners were detected in the Y2H screens (ABL1/2, PRPF40A/FBP11, RBM17/SPF45, U2AF65 and UHMK1/KIST; 3,4,20,46,48,58,59), with U2AF65 showing an expectedly high confidence score (Predicted biological score, PBS; Table 3). Several spliceosome-associated proteins and other proteins with roles in splicing were among the potential partners. However, only few proteins found in complex with SF1 by co-IP/MS were detected, suggesting that the majority of these were not direct binding partners, but co-precipitated with SF1 from HeLa cell extracts due to the numerous protein–protein interactions in the spliceosome (2).

**Table 3.** SF1 partners in Y2H screens

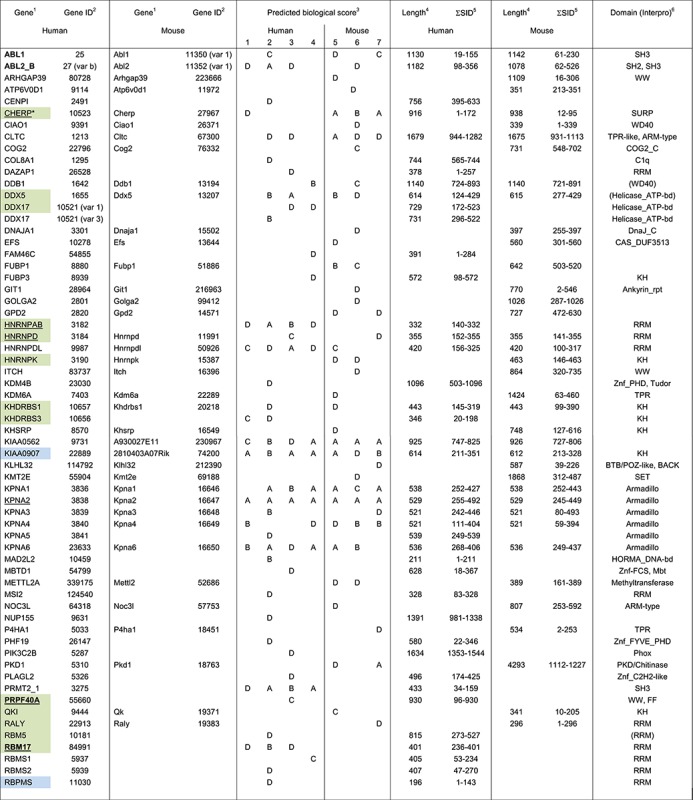									
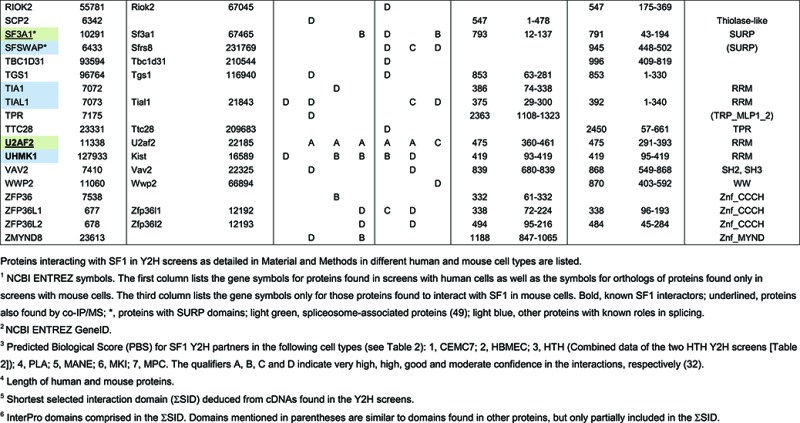									

The highest PBS was found for KPNA2 (Table 3), a subunit of the nuclear import complex. Other members of the same complex were also detected, but with a lower PBS depending on the subunit or cell type. Moreover, KIAA0907 was found as a SF1 partner with very high confidence in all cell types tested. Roles in splicing for this protein, also termed BLOM7, have been described (60). It contains a KH domain, similar to a few other proteins found in the Y2H screens with a lower PBS. Apart from these, SF1 may also directly interact with proteins containing RNA recognition motifs (RRMs) or zinc fingers. A possible relation of these proteins to SF1 function will be described in the discussion.

Among the preys with reported roles in splicing are two U2 snRNP-associated proteins: splicing factor SF3A subunit 1 (SF3A120/SF3a1) and calcium homoeostasis endoplasmic reticulum protein (CHERP; Table 3). SF3a120 is part of a heterotrimeric complex with SF3a66 and SF3a60 and essential for U2 snRNP function in spliceosome assembly (61). CHERP was originally identified as an endoplasmic reticulum protein involved in the regulation of intracellular Ca^2+^ homoeostasis (62), but is also loosely associated with the U2 snRNP, and has been reported to localize to the nucleus and function in alternative splicing (50,63,64).

**Figure 1.**
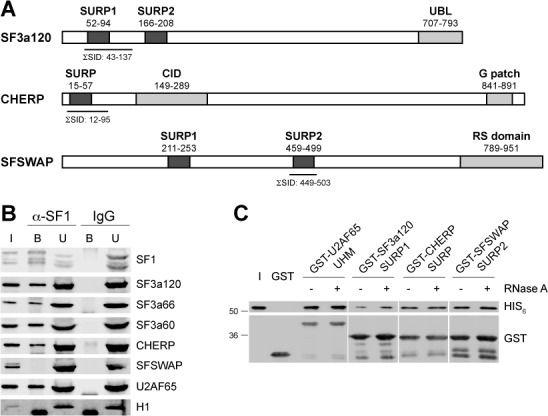
Analysis of the interaction of SF1 with SURP domain-containing proteins. (**A**) Scheme of SURP domain-containing proteins identified in Y2H screens. The domain structure of SF3a120, CHERP and SFSWAP is shown with SURP domains indicated in dark grey. Other domains are shown in light grey: CID, RNA polymerase II-binding domain; G-patch, G-patch domain; RS domain, Arg/Ser-rich domain; UBL, ubiquitin-like domain. Numbering was taken from UniProt entries (www.uniprot.org). The smallest selected interaction domain (ΣSID) deduced from cDNAs found in the Y2H screens is indicated below the proteins (numbering according to human proteins). (**B**) Co-IP. HeLa cell nuclear extract was incubated with Dynabeads Protein G coated with anti-SF1 or control IgG. Input (I; 10% of total), bound (B) and unbound (U) fractions were separated by 7.5% SDS-PAGE (10% for anti-H1) followed by western blotting with antibodies against the proteins indicated on the right side of each panel. (**C**) GST pull-down. His_6_-tagged SF1-C370 was incubated with GST alone, GST-tagged U2AF65-UHM, SF3a120-SURP1, CHERP-SURP or SFSWAP-SURP2 bound to glutathione-agarose as indicated above the figure. GST-tagged proteins were mock-treated (−) or digested with RNase A (+) as shown. The His_6_-SF1-C370 input (I; 10% of total) and bound proteins were separated by 10% SDS-PAGE and western blotted with anti-His_6_ (top) and anti-GST antibodies (bottom). The migration of protein markers is indicated in kDa on the left.

Inspection of cDNA fragments of these proteins recovered in the Y2H screens indicated that suppressor of white-apricot/Prp21 (SURP) domains (65) may mediate the interaction with SF1 (Figure 1A, Supplementary Tables S2–S9 and Supplementary Figures S4–S11). SURP domains are ≈40 amino acids long, often arranged in tandem and all known SURP domain-containing proteins are involved in splicing (66). Thus far, only the function of the second SURP domain of SF3a120 as a protein–protein interaction module has been established (66–68). The Y2H screens identified SURP1 of SF3a120 and the single SURP domain of CHERP as potential SF1 binding domains (Figure 1A and Supplementary Figures S4–S11). Moreover, the suppressor of white-apricot homolog (SFSWAP/SFRS8), an alternative splicing factor with two SURP domains (65,69–72) was detected in the Y2H screens (Table 3), with its second SURP domain potentially interacting with SF1 (Figure 1A and Supplementary Figures S4–S11). Two additional proteins containing SURP domains were found by co-IP (Table 1), the U2 snRNP-associated SURP motif-containing protein (U2SURP/SR140) and the A complex component SURP and G-patch domain-containing protein 1 (SUGP1/SF4; 50,73).

Together, these data suggest that SF1 binds U2 snRNP-associated proteins and SFSWAP via SURP domain-mediated interactions.

### SF1 directly binds SURP domains of SF3a120, CHERP and SFSWAP

To validate binding of SF1 to SURP domains, we first performed co-IPs with anti-SF1 coupled to Dynabeads Protein G from RNase A-treated HeLa cell nuclear extracts and western blotting with antibodies against the potential interacting proteins. Figure 1B shows that SF3a120, as well as SF3a60 and SF3a66 were co-precipitated with SF1; thus, the entire SF3a heterotrimer was bound. CHERP was also found in the precipitate; however, SFSWAP was not detected. U2AF65, known to interact with SF1 (4,20) and used as a positive control, was bound to SF1, whereas histone H1, a negative control, was not. None of the proteins were bound to Dynabeads Protein G coated with non-specific IgG. These results show that SF3a120 and CHERP can be co-precipitated with SF1 from HeLa cell extracts, whereas an interaction with SFSWAP was not detected.

To determine whether SF1 binding to SF3a120, CHERP and SFSWAP was mediated by direct interactions with the SURP domains identified in the Y2H screens, we performed GST pull-down assays with GST-tagged SURP domains and recombinant His_6_-tagged SF1-C370 (containing amino acids 1–370, i.e. the region common to all SF1 isoforms). His_6_-SF1-C370 was bound by the SURP domains of SF3a120, CHERP and SFSWAP, as well as by the positive control, GST-U2AF65-UHM, but not by GST alone (Figure 1C). The interactions were insensitive to RNase A treatment prior to GST pull-down, demonstrating that SURP domain binding of SF1 was not mediated by RNA. Thus, SURP1 of SF3a120, the single SURP domain of CHERP and SURP2 of SFSWAP are sufficient for direct SF1 binding, confirming the results of the Y2H screens.

### An evolutionarily conserved domain in SF1 is essential for SURP domain binding

**Figure 2.**
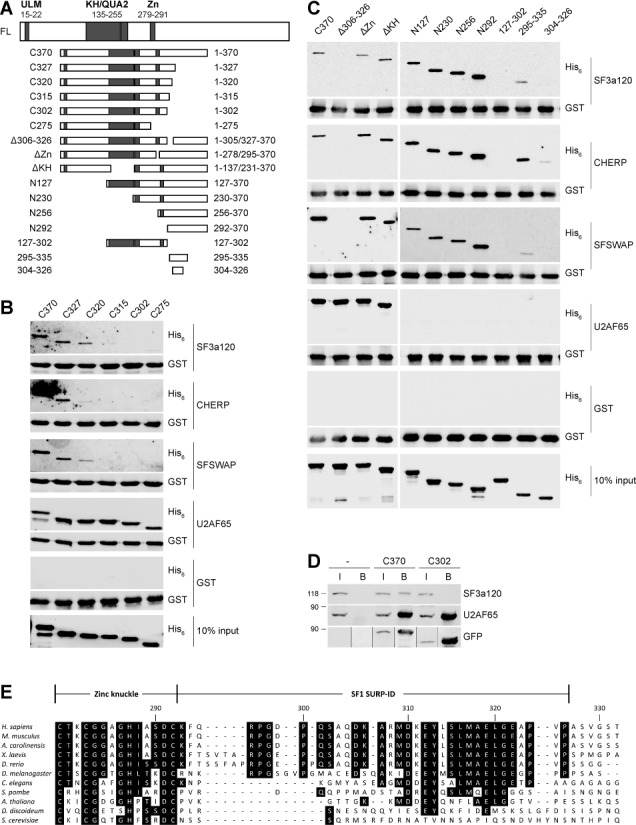
Determination of the SURP-ID of SF1. (**A**) Scheme of mutant SF1 proteins. Boxes represent known protein domains: ULM, UHM ligand motif; KH/QUA2, K-homology/Quaking2 domain; Zn, zinc knuckle. Numbering above full-length SF1 refers to amino acids. The names of SF1 mutants are shown on the left; numbers on the right refer to the residues comprising the proteins. (**B**) and (**C**) GST pull-down of mutant SF1 proteins with SURP domains. GST-tagged SF3a120-SURP1, CHERP-SURP, SFSWAP-SURP2, U2AF65-UHM and GST alone, bound to glutathione agarose (as indicated on the right), were incubated with mutant His_6_- (B) or His_6_-MBP-tagged (C) SF1 proteins as indicated above the figures. Bound proteins were separated by 10% SDS-PAGE and western blotted with anti-His_6_ (top panels) and anti-GST (bottom panels). The input (10% of the total) is shown at the bottom. (**D**) Co-IP. GFP-tagged SF1-C370 and -C302 were transiently expressed in HeLa cells. Total cell lysates were RNase A-treated and incubated with Dynabeads Protein G-coupled anti-GFP. Input (I; 0.5% of the total) and bound proteins (B) were separated by 7.5% SDS-PAGE and western blotted with anti-SF3a120, anti-U2AF65 and anti-GFP as indicated on the right. The migration of protein markers (in kDa) is shown on the left. (**E**) Multiple sequence alignment of SF1 proteins. SF1 sequences taken from the UniProt database (www.uniprot.org) were aligned with ClustalW2 (www.ebi.ac.uk/Tools/msa/clustalw2/). The region from the zinc knuckle of human SF1 and 40 amino acids C-terminal of this domain is shown. Numbering is given for human SF1. Amino acids identical in more than 50% of the sequences are marked.

The region of SF1 responsible for the SURP domain interaction was analyzed in GST pull-down assays with His_6_-tagged C- and N-terminal as well as internal deletion mutants of SF1 (Figure 2A). Compared to SF1-C370, binding to GST-SF3a120-SURP1, CHERP-SURP and SFSWAP-SURP2 was slightly weakened with SF1-C327 and strongly reduced upon deletion to amino acid 320 (Figure 2B). Binding of SF1-C315 was barely detectable and further deletion of SF1 residues completely abolished the SURP domain interaction. Thus, the C-terminal border of the SURP interaction domain (ID) lies between SF1 residues 315 and 327.

SF1 mutants with N-terminal His_6_ and maltose-binding protein (MBP) tags were used to further delimit the SF1 SURP-ID. N-terminal deletions up to, and including the zinc knuckle (N127 to N292; Figure 2C) did not abolish binding of SF1 to the SURP domains. Thus, neither the SF1 KH/QUA2 domain nor the zinc knuckle are involved in the interaction, which was confirmed with SF1 mutants carrying internal deletions of these domains (ΔKH and ΔZn). SF1 residues 295–335 were barely sufficient for the interaction and amino acids 127–302 or 304–326 did not bind the SURP domains. Moreover, an internal deletion of residues 306–326 abolished binding. The C-terminal or internal deletions did not affect binding to GST-U2AF65-UHM, used as a positive control, whereas none of the N-terminal deletions interacted with GST-U2AF65-UHM, since the U2AF ligand motif (ULM) of SF1 was not present. Binding to GST alone was not observed. Together, these data indicate that the region between residues 293 and 327 of SF1 is required for the interaction with SURP domains.

To further test the relevance of this domain for SURP binding, GFP-tagged SF1-C370 and -C302 were transiently expressed in HeLa cells. RNase A-treated whole cell lysates were immunoprecipitated with Dynabeads Protein G-coupled anti-GFP. SDS-PAGE and western blotting with anti-SF3a120 showed that endogenous SF3a120 interacted with GFP-SF1-C370 but not -C302, whereas U2AF65 bound both proteins (Figure 2D). Thus, the SF1 SURP-ID identified *in vitro* with recombinant proteins is also essential for binding SF3a120 in HeLa cell lysates.

A multiple sequence alignment of SF1 revealed the presence of a region well-conserved from *Drosophila* to humans (amino acids 293–326 of human SF1; Figure 2E) encompassing the residues that eliminated the SURP domain interaction. This region is highly conserved in mammals, *Xenopus* and zebrafish, and partial conservation of key residues (amino acids 301–321) is seen in other organisms, including *Arabidopsis*, *Schizosaccharomyces pombe* and *Dictyostelium*, with the exception of *S. cerevisiae*.

From these results we conclude that an evolutionarily conserved domain in SF1, spanning amino acids 293–327 is essential for binding SURP domains.

### The SF1/SURP interaction is required for efficient early spliceosome assembly

The above results demonstrate that SF1 can bind the U2 snRNP through direct interactions with SF3a120 and CHERP. SF1 recognizes the BPS in complex E and the U2 snRNP is weakly bound to this complex before base pairing of the U2 snRNA to the BPS and pre-mRNA binding of several U2 snRNP-associated proteins in complex A (5,10,13,56,57). To test the possibility that SF1 is involved in the recruitment of the U2 snRNP to the pre-mRNA, we compared spliceosome formation in HeLa cell nuclear extracts and SF1-depleted extracts in the absence or presence of recombinant SF1 proteins with or without the SURP-ID. To facilitate quantification of the results, spliceosome assembly was analyzed with an AdML pre-mRNA lacking the 5′ splice site and containing 75 nts of the 3′ end of intron 1 and part of exon 2. Similar splicing substrates are efficiently assembled into a 3′ splice site complex related to pre-splicing complex A (termed A3’ complex hereafter) but not converted to complex B (43). In addition, the original BPS of AdML intron 1 was changed to the yeast consensus BPS, UACUAAC, which is the optimal BPS for mammalian splicing (74) and also the preferred binding site of SF1 (5,25,75).

**Figure 3.**
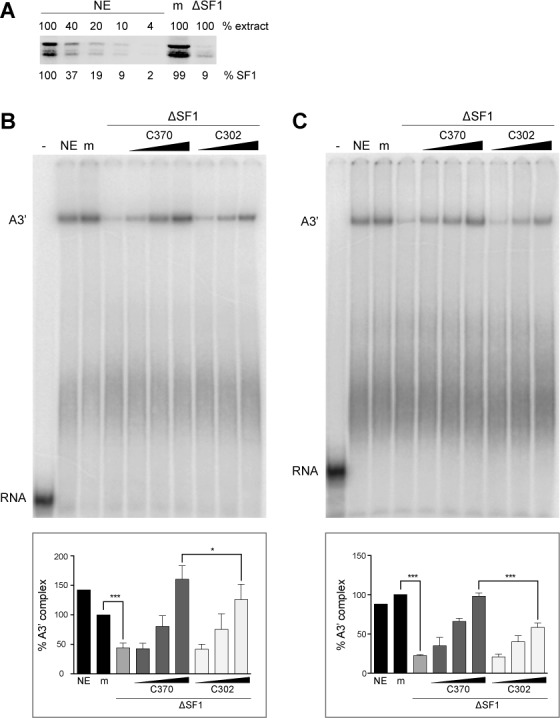
Deletion of the SF1 SURP-ID negatively affects spliceosome assembly. (**A**) SF1 immunodepletion. HeLa nuclear extract (NE) was depleted by incubation with Dynabeads Protein G-coupled anti-SF1. Serial dilutions of untreated (NE), mock-depleted (m) and SF1-depleted extracts (ΔSF1) were separated by 7.5% SDS-PAGE and western blotted with anti-SF1. The intensities of SF1 isoforms (% SF1) were quantified with the Odyssey Fc imaging system (LI-COR Biosciences) and normalized to 100% untreated nuclear extract. (**B**) and (**C**) Spliceosome assembly. Splicing reactions were performed in triplicate at 30°C for 30 min with AdML 3′ splice site pre-mRNAs containing a consensus (B) or weak BPS (C) in the absence (−) or presence of untreated (NE), mock- (m) or SF1-depleted (ΔSF1) HeLa nuclear extracts. Reactions containing SF1-depleted extract were supplemented with increasing amounts of His_6_-SF1-C370 or -C302 (0.022, 0.22 or 2.2 pmol) as indicated. Reaction products were separated in native 4% polyacrylamide gels, which were dried and exposed to PhosphorImager screens. A representative gel is shown on the top. Quantification of the results is shown on the bottom. ‘% A3’ complex’ represents the ratio of A3’ complex to total lane intensities. Values were normalized to mock-treated extract. *P < 0.04, ***P < 0.006 (Student's unpaired *t*-test; *n* = 3); error bars indicate standard error of the mean (SEM).

Immunodepletion with anti-SF1 bound to Dynabeads Protein G resulted in a 91% reduction of different SF1 isoforms compared to untreated or mock-depleted nuclear extracts (Figure 3A). Other proteins, such as U2AF65 or SF3a120, were not co-depleted (Supplementary Figure S2A). A3’ complex formation tested by incubation for 30 min at 30°C in triplicate experiments was reduced to an average of 44% of that seen in the mock-depleted extract (Figure 3B). The SF1-depleted extract was then complemented with equivalent concentrations of His_6_-SF1-C370 or -C302 (Supplementary Figure S3), containing or lacking the SURP-ID, respectively (Figure 2A and B). Addition of SF1-C370 rescued A3’ complex formation to 160% compared to mock-depleted extract in a dose-dependent manner, whereas complex formation in the presence of SF1-C302 was less efficient (126%; Figure 3B). The observation that the recombinant proteins rescued A3’ complex assembly to more than 100% could be due to the fact that their concentration exceeded that of endogenous SF1. Comparison of protein levels of endogenous and recombinant proteins is difficult, because endogenous SF1 exists in multiple isoforms (Figure 3A; 76) and their exact contribution to splicing is not established.

A rather modest effect of SF1 depletion on spliceosome formation, despite an at least 98% reduction in SF1 protein has been reported by Guth and Valcárcel (24). The same authors showed that the effect of SF1 depletion was more pronounced with a pre-mRNA containing a weak as compared to a strong BPS. Similarly, depletion of SF1 in yeast causes more severe splicing defects with reporters containing mutated splice sites (14). We therefore repeated the experiment with the same pre-mRNA as above, but containing the weak IgM BPS (AAUUCAC; 24,77). Compared to mock-treated extract, SF1 depletion reduced complex formation to about 23% (Figure 3C). Complementation with SF1-C370 rescued A3’ complex formation to nearly mock-depleted levels (98%), whereas addition of SF1-C302 resulted in a significantly reduced rescue of 58%. Thus, the region of SF1 necessary for the interaction with U2 snRNP proteins is also required for efficient A3’ complex assembly. The more marked effect seen with the pre-mRNA containing a suboptimal BPS can be explained by the fact that the consensus BPS is the preferred SF1 binding site (5,25,75).

**Figure 4.**
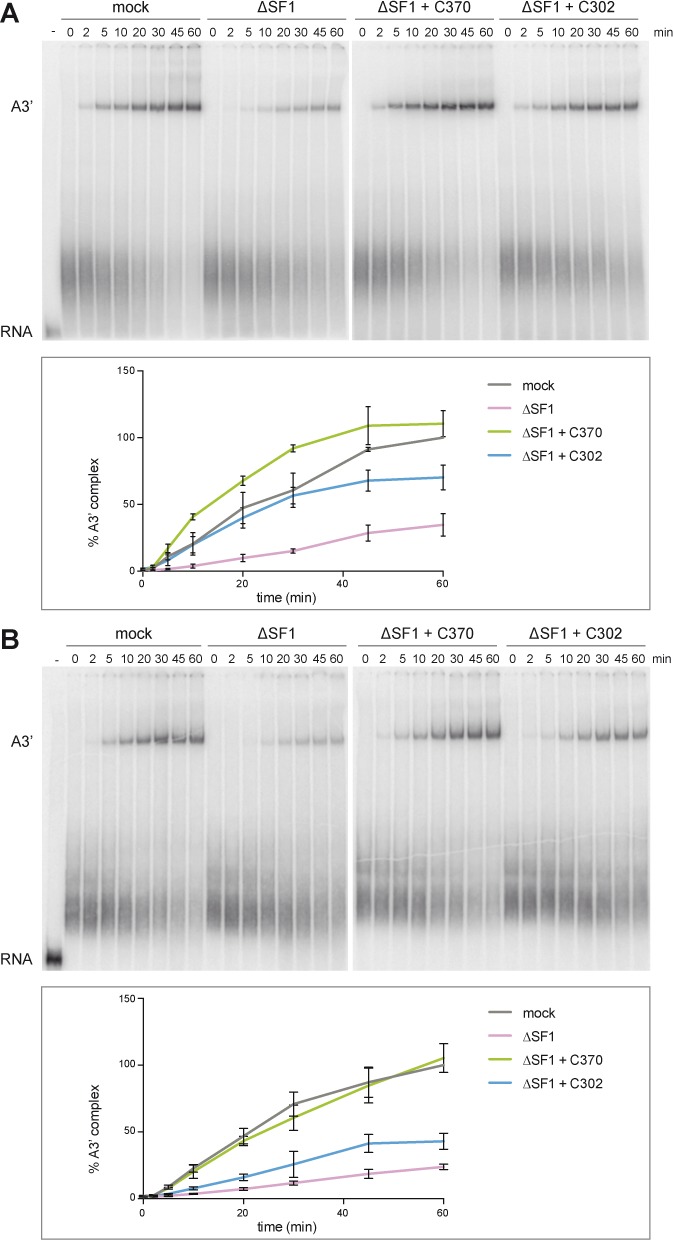
Deletion of the SF1 SURP-ID affects the kinetics of spliceosome assembly. Splicing reactions with AdML 3′ splice site pre-mRNAs containing a consensus (**A**) or weak BPS (**B**) were performed in triplicate at 30°C for the times indicated in the presence of mock- or SF1-depleted (ΔSF1) nuclear extract supplemented with 2.2 pmol His_6_-SF1-C370 or -C302 as shown. Samples were separated in native 4% polyacrylamide gels, which were dried and exposed to PhosphorImager screens. Representative gels are shown in the top panels. The results are quantified in the bottom panels. ‘% A3’ complex’ represents the ratio of A3’ complex to total lane intensities. Values were normalized to those of mock-treated extracts at the 60-min time points. Error bars indicate SEM.

Previous studies suggested a kinetic role for SF1 in spliceosome assembly (14,24). The above results indicate that SF1 binds two U2 snRNP proteins and the SF1 SURP-ID is required for efficient A3’ complex formation. To test whether the SF1-U2 snRNP interaction underlies the kinetic role of SF1, we performed a time course of A3’ complex formation. Pre-mRNAs with a consensus or weak BPS were incubated with mock- or SF1-depleted HeLa nuclear extract supplemented with His_6_-SF1-C370 or -C302. Representative results of experiments performed in triplicate are shown in the top panels of Figure 4A and B. A3’ complex formation was quantified and normalized to the values for the 60-min time points of the mock-depleted extract (bottom panels). SF1 depletion resulted in a 65 or 76% reduction in A3’ complex assembly with pre-mRNAs containing a consensus or weak BPS, respectively. Addition of SF1-C370 rescued complex formation to the levels of mock-depleted extract with both pre-mRNAs. When reactions were complemented with SF1-C302, A3’ complex formation with the consensus BPS substrate was reduced only at the 45 and 60-min time points, plateauing at 70%. In contrast, A3’ complex formation performed in the presence of SF1-C302 and the pre-mRNA with a weak BPS was less efficient at all time points, with a maximum level of 43% at 60 min. Thus, the interaction between SF1 and the U2 snRNP is essential for efficient A3’ complex formation, especially when U2 snRNA/BPS base pairing is suboptimal. This observation can explain the previously reported kinetic effect on spliceosome assembly (24).

### The absence of the SF1 SURP-ID does not compromise U2AF65 binding to the RNA

**Figure 5.**
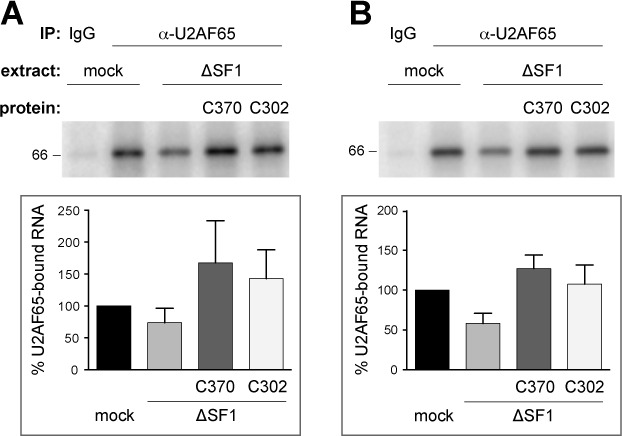
Deletion of the SF1 SURP-ID does not reduce U2AF65 binding to the pre-mRNA. (**A**) and (**B**) U2AF65 UV cross-linking to AdML 3′ splice site substrates. Splicing reactions containing radio-labeled RNA with a consensus (A) or weak (B) BPS, mock or SF1-depleted (ΔSF1) extracts complemented with 2.2 pmole His_6_-SF1-C370 or -C302 as indicated were incubated at 30°C for 15 min. Samples were UV cross-linked, RNase A-treated and immunoprecipitated with control IgG or anti-U2AF65, as indicated. RNA–protein complexes were separated by 10% SDS-PAGE. Gels were dried and exposed to PhosphorImager screens. The top panels show representative results of triplicate experiments; quantifications are shown in the bottom panels. ‘% U2AF65-bound RNA’ indicates the percentage of the intensity of the cross-linked RNAs normalized to the RNA immunoprecipitated with anti-U2AF65 from mock-treated extract. Data are shown as mean value ± SEM.

SF1 and U2AF65 cooperatively bind the pre-mRNA and increase each others affinity for RNA (3). Thus, SF1 depletion also causes decreased binding of U2AF65 to the polypyrimidine tract with a concomitant decrease in spliceosome assembly (24), which likely contributes to the lower levels of A3’ complex formation seen above. To rule out the possibility that RNA binding of U2AF65 is affected in a SF1-depleted extract complemented with recombinant SF1 proteins, we performed UV cross-linking of U2AF65 in 15-min reactions containing mock- or SF1-depleted extracts and pre-mRNAs with a consensus or weak BPS. Triplicate samples were UV cross-linked, RNase A-treated and incubated with Dynabeads Protein G-coupled control IgG or anti-U2AF65. Bound material was analyzed by SDS-PAGE and the fraction of RNA bound to U2AF65 was quantified (Figure 5). U2AF65 efficiently cross-linked to both pre-mRNAs in the mock-depleted samples. Only background cross-linking was detected in IPs performed with control IgG. As expected from previous results (24), pre-mRNA binding of U2AF65 was partially reduced upon SF1 depletion. However, addition of either His_6_-SF1-C370 or -C302 increased U2AF65/RNA binding to the levels seen in mock-depleted extract, consistent with the fact that both proteins contain the ULM and thus can bind U2AF65 (Figure 2B). We therefore conclude that the reduced levels of A3’ complex formation observed in SF1-depleted extracts complemented with SF1-C302 are due to the lack of interaction of SF1 with the U2 snRNP and not caused by decreased pre-mRNA binding of U2AF65.

## DISCUSSION

Results from co-IP/MS experiments and Y2H screens have provided information about novel and, in part, unexpected partners of SF1. The co-IP/MS experiment demonstrated that about half of the proteins co-precipitated with SF1 from HeLa cell nuclear extracts are involved in early steps of spliceosome assembly, confirming an early role for SF1 in splicing (19,20). Surprisingly, most of the U2 snRNP-associated proteins were co-precipitated with SF1. Y2H screens revealed potential interactions of SF1 with the SURP domain-containing, U2 snRNP-associated proteins SF3a120 and CHERP, as well as the alternative splicing factor SFSWAP. Direct binding of SF1 to SURP domains of the three proteins were confirmed in GST pull-down assays and the interaction domain was mapped to an evolutionarily conserved, central region of SF1. We furthermore show that the SF1/U2 snRNP interaction is necessary for efficient spliceosome assembly, suggesting a role for SF1 in the early recruitment of the U2 snRNP to the pre-mRNA.

### A novel role for SF1 in U2 snRNP recruitment

Consistent with a role for SF1 at the onset of spliceosome assembly (19,20), anti-SF1 antibodies precipitated more than half of the proteins present in early splicing complexes, compared to only 8% of proteins associated with later complexes or functioning in splicing catalysis and post-splicing events (Table 1; 2,49). However, although only five of 18 annotated A complex components were co-precipitated, almost all U2 snRNP-associated proteins were present. This finding was unexpected, since the events leading to stable pre-mRNA binding of the U2 snRNP at the time of A complex assembly are thought to trigger the release of SF1 at the same time. In *S. cerevisiae* SF1 (BBP) was detected in the ATP-independent commitment complex 2, but not the pre-spliceosome (14). In the human system proteins in the ≈80-kDa range, most likely representing SF1 isoforms, cross-link to the BPS in a U1 snRNP-dependent and ATP-independent manner at very early times of spliceosome assembly (78). These cross-links disappear in the presence of ATP and are replaced by cross-links to proteins of 14, 35 and 150 kDa, which persist in complex A. The 14-kDa protein is SF3b14a/p14, a U2 snRNP protein directly binding to the BPS adenosine (11,12,78). The 150-kDa protein probably represents SF3b155, a U2 snRNP component binding upstream and downstream of the BPS and interacting with the U2AF65-UHM, the same site occupied by SF1 (10,51,53). In addition, a ‘U2 mimic’, an oligoribonucleotide base-pairing to the BPS and neighboring sequences, prevents BPS binding of SF1 (3). Finally, SF1 has only been detected in small amounts in A complexes isolated from HeLa cells under physiological conditions and the material used could have contained small amounts of E complexes (79). These findings imply that the SF1/U2 snRNP interaction occurs prior to A complex formation. Since the U2 snRNP has been detected in complex E (55–57), our data strongly suggest that SF1, binding to the SURP domains of SF3a120 or CHERP, aids the initial recruitment of the U2 snRNP to the assembling spliceosome.

We envision the following series of events: SF1 and U2AF65 interact with one another, involving the ULM at the N terminus of SF1 and the UHM of U2AF65 (51). This is followed by their cooperative binding to the BPS and the polypyrimidine tract (3). SF1 binds the BPS through its KH domain (6), leaving the SURP-ID available for binding SF3a120 or CHERP. The SF1/BPS interaction pre-bulges the BPS adenosine, whereas other nucleotides are accessible for U2 snRNA base pairing (6). In principle, SF1 could interact with the U2 snRNP before or after binding to U2AF65; in either case it would position the U2 snRNP close to the BPS. The transition to complex A then occurs through replacement of the SF1/U2AF65 interaction by binding of SF3b155 to the U2AF65 UHM, binding of SF3b14a/p14 to the BPS adenosine, base pairing of U2 snRNA to the BPS and binding of SF3a and SF3b subunits to the pre-mRNA on both sides of the BPS, tethering the U2 snRNP for later steps (10,11,13,53,78). In this scenario, SF1 initiates U2 snRNP recruitment and facilitates the events required for A complex formation by placing the U2 snRNP into the vicinity of the BPS and U2AF65, whereas U2AF65 stabilizes the association of the U2 snRNP with the spliceosome once SF1 is released.

The interaction of SF1 with U2 snRNP-associated proteins is required for efficient spliceosome assembly, since A3’ complex formation is strongly reduced following SF1 depletion and can be restored by addition of SF1 containing, but less so by addition of SF1 lacking the SURP-ID (Figures 3 and 4). This effect is not due to the disruption of the SF1/U2AF65 interaction, since both SF1 proteins contain the ULM and pre-mRNA binding of U2AF65 was not compromised in the presence of either protein (Figure 5). Guth and Valcárcel (24) suggested a function of SF1 in addition to increasing the affinity of U2AF65 for the polypyrimidine tract. The results presented here strongly suggest that this additional role is the binding of SF1 to the U2 snRNP, thereby directly promoting the U2 snRNP/BPS interaction.

Although SF1 has long been considered a constitutive splicing factor, it is not required to splice all introns (22,25). In these cases, U2AF65 may be sufficient to recruit the U2 snRNP, perhaps helped by other proteins (80). Also, U2AF65 may not be necessary for the recognition of 12% of functional human 3′ splice sites (81); thus, SF1 may play a major role in the recruitment of the U2 snRNP to introns that are not bound by U2AF65.

### The interaction of SF1 with SURP domains

The Y2H screens identified SURP1 of SF3a120, the single SURP domain of CHERP and SURP2 of SFSWAP as SF1 interaction partners, which was confirmed with recombinant proteins (Table 3, Figure 1 and Supplementary Figures S4–S11). The remaining SURP domains of SF3a120 and SFSWAP could not be tested, since the bacterially expressed proteins were insoluble (data not shown). However, the following points argue that SF1 only interacts with the SURP domains tested. First, although some of the cDNAs isolated in the Y2H screens completely or partially included the other SURP domains of SF3a120 and SFSWAP, SF3a120 SURP1 and SFSWAP SURP2 were present in all cDNAs recovered. Second, Kuwasako et al. (66) only obtained SURP2 of SF3a120 in soluble form when co-expressed with SF3a60. In analogy to these experiments, co-expression of SF1 and SF3a120 SURP2 did not yield soluble SURP2 (data not shown), suggesting that SF1 does not interact with SF3a120 SURP2. Third, SURP domains can be classified into two evolutionarily related subgroups (66). The SURP domains of SF3a120, SFSWAP and CHERP isolated in the SF1 Y2H screens belong to subgroup 1, whereas SF3a120 SURP2 and SFSWAP SURP1 belong to subgroup 2. It is therefore highly likely that SF1 only interacts with SURP domains of subgroup 1, but not those of subgroup 2.

The SF1 SURP-ID is located immediately C-terminal to the zinc knuckle (Figure 2). Its sequence is almost invariant in mammals and conserved to a lesser extent in *A. thaliana* and *S. pombe*, but it is not present in *S. cerevisiae* SF1. BLAST searches did not identify a related sequence in other mammalian proteins. SF3a60 binds SURP2 of SF3a120 and single amino acid changes in SURP2 and SURP1 swap the identity of the domains, i.e. prevent SURP2 binding of SF3a60 but allow for SURP1 binding (66). It is therefore intriguing to speculate that the interaction sites in SF3a60 and SF1 show a certain degree of similarity. A sequence alignment between the SF1 and SF3a60 SURP-IDs does not reveal obvious homology (data not shown). The SF3a60 SURP-ID forms a long amphipathic α-helix (66) and an equivalent interaction surface has been described between the *S. cerevisiae* orthologs (68). Analysis with the Phyre2 protein fold recognition server (82) also predicts a helical structure for the SF1 SURP-ID (data not shown). Differences in the mode of interaction would however be expected, since SF3a forms a stable heterotrimer (41), whereas the SF1/SF3a120 interaction is dynamic and resolved at the time of A complex assembly. Future structural studies should define similarities and differences in the binding modes of SF3a60 and SF1 to SF3a120.

### Lack of conservation of the SF1 SURP-ID in *S. cerevisiae*

Somewhat as a surprise, the SF1 SURP-ID is not conserved in *S. cerevisiae* SF1 (BBP). In addition, SURP1 of the yeast SF3a120 ortholog Prp21p lacks four amino acids close to a 3_10_ helix present in the human protein (66), which could change the spatial organization of the interaction site. This may suggest that BBP and Prp21p do not interact. In fact, Y2H interactions between BBP and Prp21p have not been detected (J.-C.R., unpublished results) and are also not documented in the *Saccharomyces* Genome Database (http://www.yeastgenome.org/).

Could the BBP/Prp21p interaction be dispensable in *S. cerevisiae*? The vast majority of yeast introns contain the consensus BPS, UACUAAC, (83) with a perfect complementarity to the BPS-interacting sequence of U2 snRNA (G-UAGUA; the dash indicates the missing complementarity to the bulged-out BPS adenosine). Given our observation that the SF1/U2 snRNP interaction is more important for efficient splicing of introns with a weak BPS, SF1 may thus not be required for U2 snRNP recruitment in yeast.

### The SF1/SFSWAP interaction

SFSWAP was identified as an additional partner of SF1 in Y2H screens from mouse tissues. SF1 binding to its SURP2 domain was confirmed in GST pull-down assays, but we did not detect the interaction by anti-SF1 IP from HeLa cell lysates. SFSWAP is an alternative splicing regulator that also autoregulates the splicing of its own pre-mRNA (65,69–72); however, targets of its regulation remain largely unknown. Given that SF1 also acts as an alternative splicing factor (25–28), the two proteins may physically interact to regulate the splicing of perhaps only a few pre-mRNAs or only in certain tissues, which could explain the failure to detect the interaction in HeLa cells. Finding common splicing substrates with appropriate methods could solve this question.

### Additional potential partners of SF1

Anti-SF1 antibodies co-precipitated only few proteins without a reported connection to splicing (Table 1). Some of these are involved in transcription, which may be related to the published role for SF1 in transcription repression (84,85). Similarly, several transcription factors were identified as potential partners of SF1 in the Y2H screens (e.g. zinc finger-containing proteins).

The Y2H screens were informative concerning other potential SF1 partners and structural domains mediating interactions with SF1 (Table 3). KPNA2 was found as a prey in all cell types tested with the highest-confidence PBS observed. Other members of this family of nuclear import factors were also detected, but with lower PBS. SF1 is a nuclear protein and therefore its interaction with the nuclear import machinery may not be surprising. However, Y2H screens with other nuclear proteins did not find KPNAs in such abundance (J.-C.R., unpublished data). The shortest selected interaction domain (ΣSID) of the KPNAs comprises armadillo repeats, which are also found in the nuclear pore complex protein Nup155, another prey of SF1. Therefore, SF1 may have a thus far unknown function related to nuclear import.

Another protein with a high PBS found in all cell types analyzed is KIAA0907/BLOM7. It was initially identified in Y2H screens with a component of the human Prp19 complex and has been shown to increase splicing in HeLa cell nuclear extracts and regulate alternative splice site decisions (60). BLOM7 contains two KH domains, the second of which is comprised in the SF1 ΣSID. Six additional proteins with KH domains covered by the ΣSID were found as SF1 preys (KHDRBS1/SAM68; KHDRBS3/SLM-2; KHSRP/FUBP2; hnRNPK; FUBP3; QKI), most of which play roles in splicing (86–90). Interestingly, SF1 itself contains a KH domain of the same type as those of KHDRBS1, KHDRBS2, QKI and BLOM7.

KIAA0562/CEP104 is a centrosomal protein of 104 kDa and another example of a prey identified in all cell types with a high PBS. The ΣSID of CEP104 does not comprise any known structural domain and a potential role for a SF1/CEP104 interaction is elusive.

In addition to these proteins, SF1 preys include RRM-containing proteins, most of which with reported functions in splicing. Interestingly, the ΣSIDs of these proteins often comprise tandem RRMs. This observation may suggest that SF1 not only binds proteins with non-canonical RRMs of the UHM class (47,48,51,91), but also other types of RRMs. Taken together, the Y2H screens have provided a vast amount of information about possible SF1 partners and novel interaction domains, the relevance of which remains to be investigated.

To conclude, our data demonstrate a new role for SF1 in the recruitment of the U2 snRNP to the spliceosome by interaction with SURP domain-containing proteins and the potential to regulate alternative splicing events through a SURP domain-mediated interaction with SFSWAP. Our findings moreover stress the function of SURP domains as protein–protein interaction modules and future studies may identify additional proteins interacting with SURP domains. Furthermore, the Y2H screens identified potential novel partners of SF1. Studying these interactions may reveal additional functions of SF1 in splicing and other cellular pathways.

## Supplementary Material

SUPPLEMENTARY DATA
