# Supplementary material for: Mammalian splicing factor SF1 interacts with SURP domains of U2 snRNP-associated proteins
Source: Nucleic Acids Res. 2015 Sep 29;43(21):10456–73. doi: 10.1093/nar/gkv952 (PMC4666396; doi:10.1093/nar/gkv952)
Supplement: SUPPLEMENTARY DATA [file supp_43_21_10456__index.html]

Mammalian splicing factor SF1 interacts with SURP domains of U2 snRNP-associated proteins — Mammalian splicing factor SF1 interacts with SURP domains of U2 snRNP-associated proteins — SUPPLEMENTARY DATA 

# Mammalian splicing factor SF1 interacts with SURP domains of U2 snRNP-associated proteins

## SUPPLEMENTARY DATA

- SUPPLEMENTARY DATA
- SUPPLEMENTARY DATA
- SUPPLEMENTARY DATA
- SUPPLEMENTARY DATA
