# Supplementary material for: Mammalian splicing factor SF1 interacts with SURP domains of U2 snRNP-associated proteins
Source: Nucleic Acids Res. 2015 Sep 29;43(21):10456–73. doi: 10.1093/nar/gkv952 (PMC4666396; doi:10.1093/nar/gkv952)
Supplement: SUPPLEMENTARY DATA [file supp_gkv952_nar-00201-a-2015-File008.pdf]

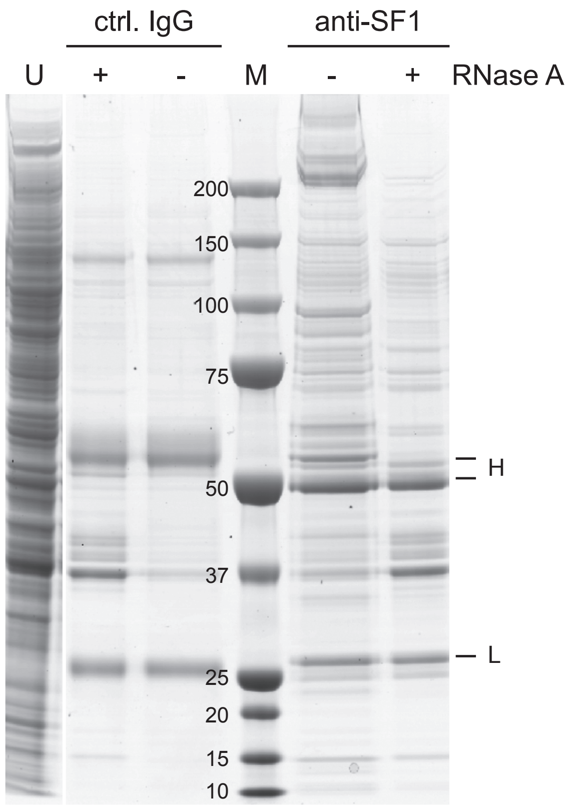


**Supplementary Figure S1.** Co-immunoprecipitation of HeLa cell proteins with anti-SF1 antibodies. A HeLa cell lysate was incubated in the absence (-) or presence (+) of RNase A followed by precipitation of proteins with control (ctrl.) IgG or anti-SF1 antibodies coupled to Protein G Sepharose, as indicated on top of the figure. Bound proteins were separated by 1D PAGE and stained with Coomassie blue. Unbound (U) proteins are shown in the first lane. The migration of marker proteins (M; in kDa) is indicated.

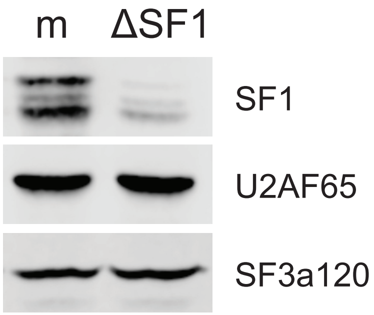


**Supplementary Figure S2.** Detection of U2AF65 and SF3a120 in SF1-depleted extracts. HeLa nuclear extract was depleted of SF1 as in Figure 3A. Twenty-µl aliquots of mock-depleted (m) and SF1-depleted extracts (ΔSF1) were separated by 7.5% SDS-PAGE and Western blotted with antibodies against SF1, U2AF65 and SF3a120.

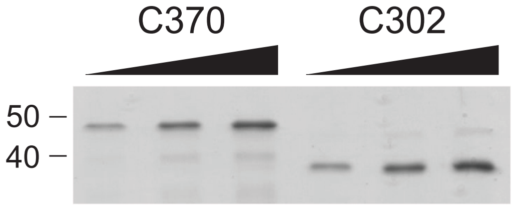


**Supplementary Figure S3.** Quantification of recombinant proteins. Increasing amounts of His6-SF1-C370 and SF1-C302 (2.2, 4.4 and 6.6 pmol) were separated by 10% SDS-PAGE followed by Coomassie blue staining. The migration of protein markers is indicated in kDa on the left.
