# Supplementary material for: Mammalian splicing factor SF1 interacts with SURP domains of U2 snRNP-associated proteins
Source: Nucleic Acids Res. 2015 Sep 29;43(21):10456–73. doi: 10.1093/nar/gkv952 (PMC4666396; doi:10.1093/nar/gkv952)

Supplementary Figures S4 - S11. In the following figures the bait fragment of SF1 and the Selected Interacting Domain (SID) of the prey proteins are aligned with functional and structural domains (PFAM, SMART, TMHMM, SignalP, Coil algorithms) of these proteins. The SID is defined as the amino acid sequence shared by all prey fragments matching the same reference protein. SIDs have been found in numerous cases to correspond to an identified structural or functional domain. The color coding for bait and SID fragments as well as for domains is shown on page 1 of all supplementary figures.

Supplementary Figure S4 - page 1

CEMC7 - Human T cell line

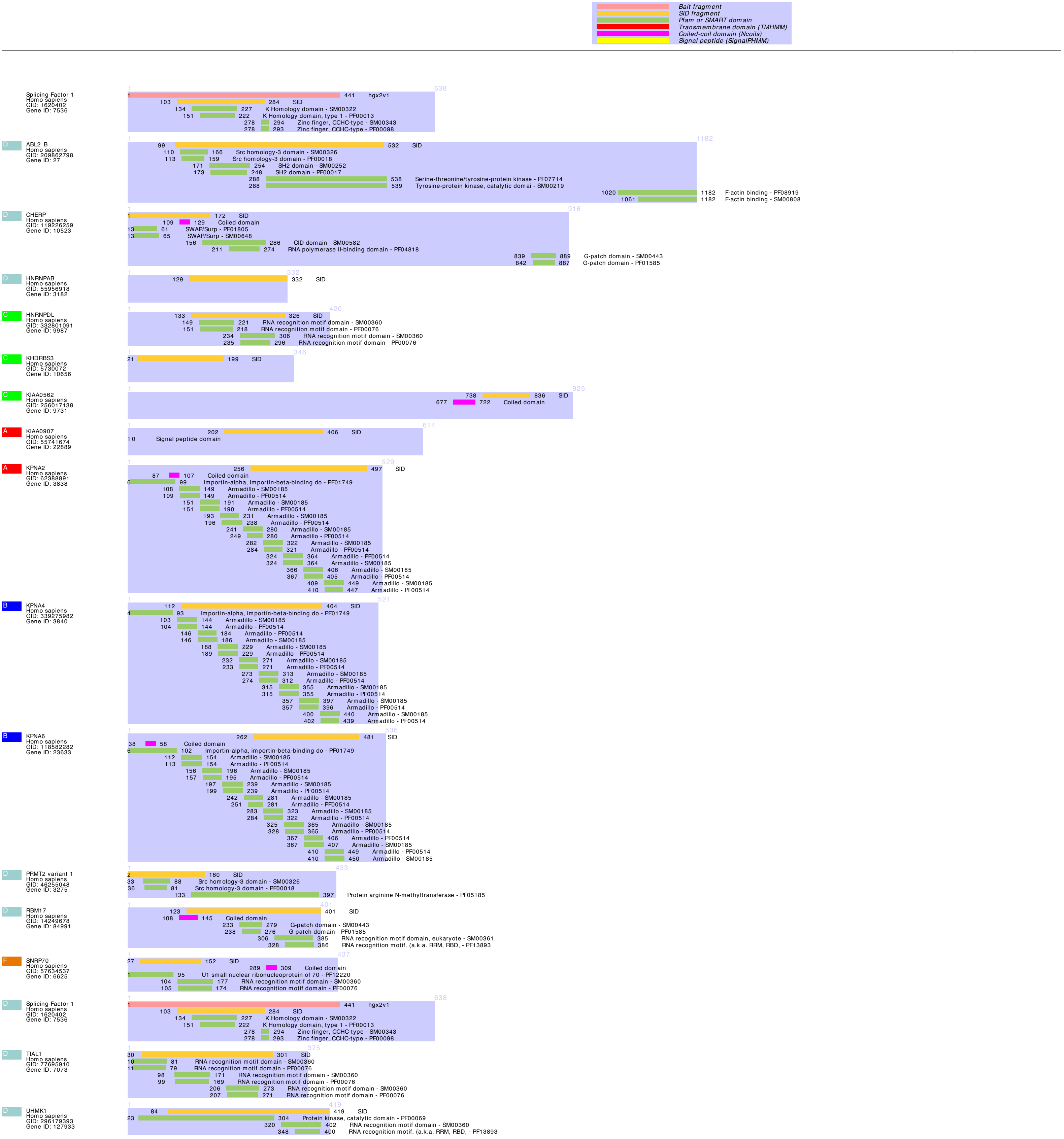

Supplementary Figure S4 - page 2

CEMC7 - Human T cell line

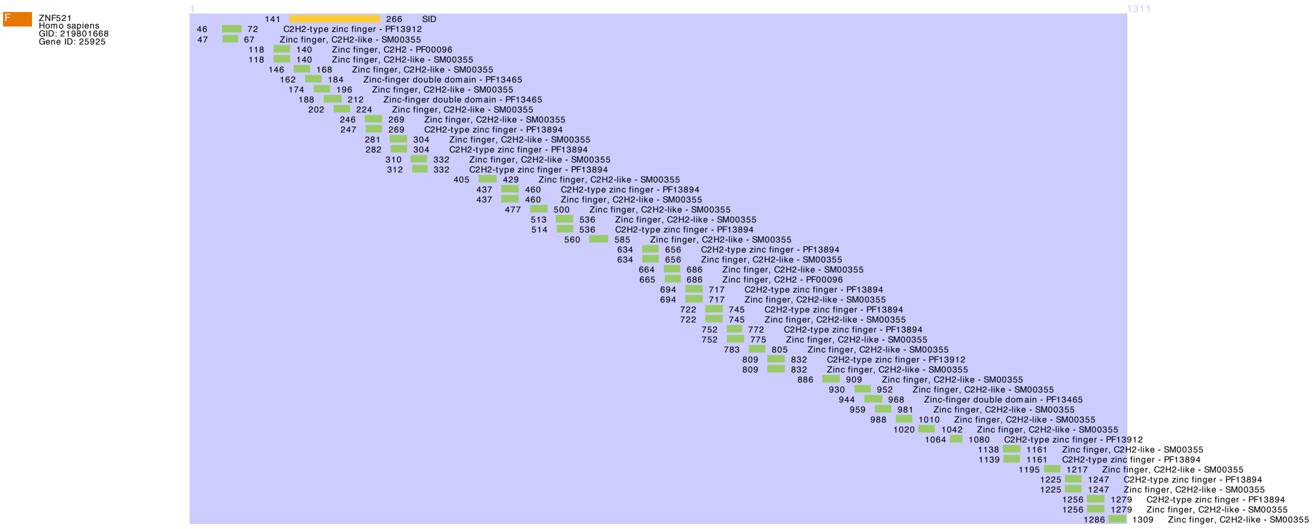

Supplementary Figure S5 - page 1

HBMEC - Human bone marrow endothelial cells

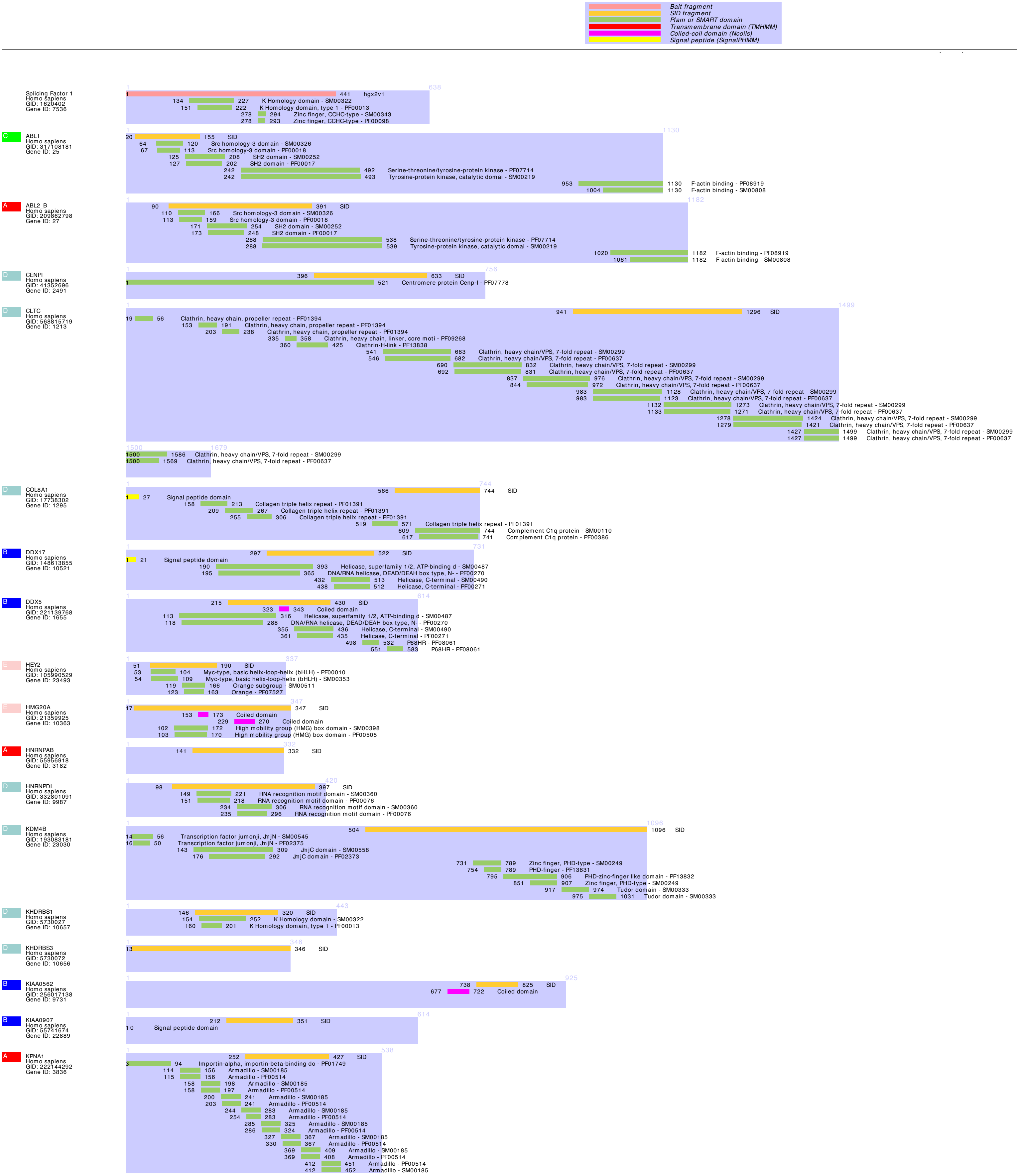

Supplementary Figure S5 - page 2

HBMEC - Human bone marrow endothelial cells

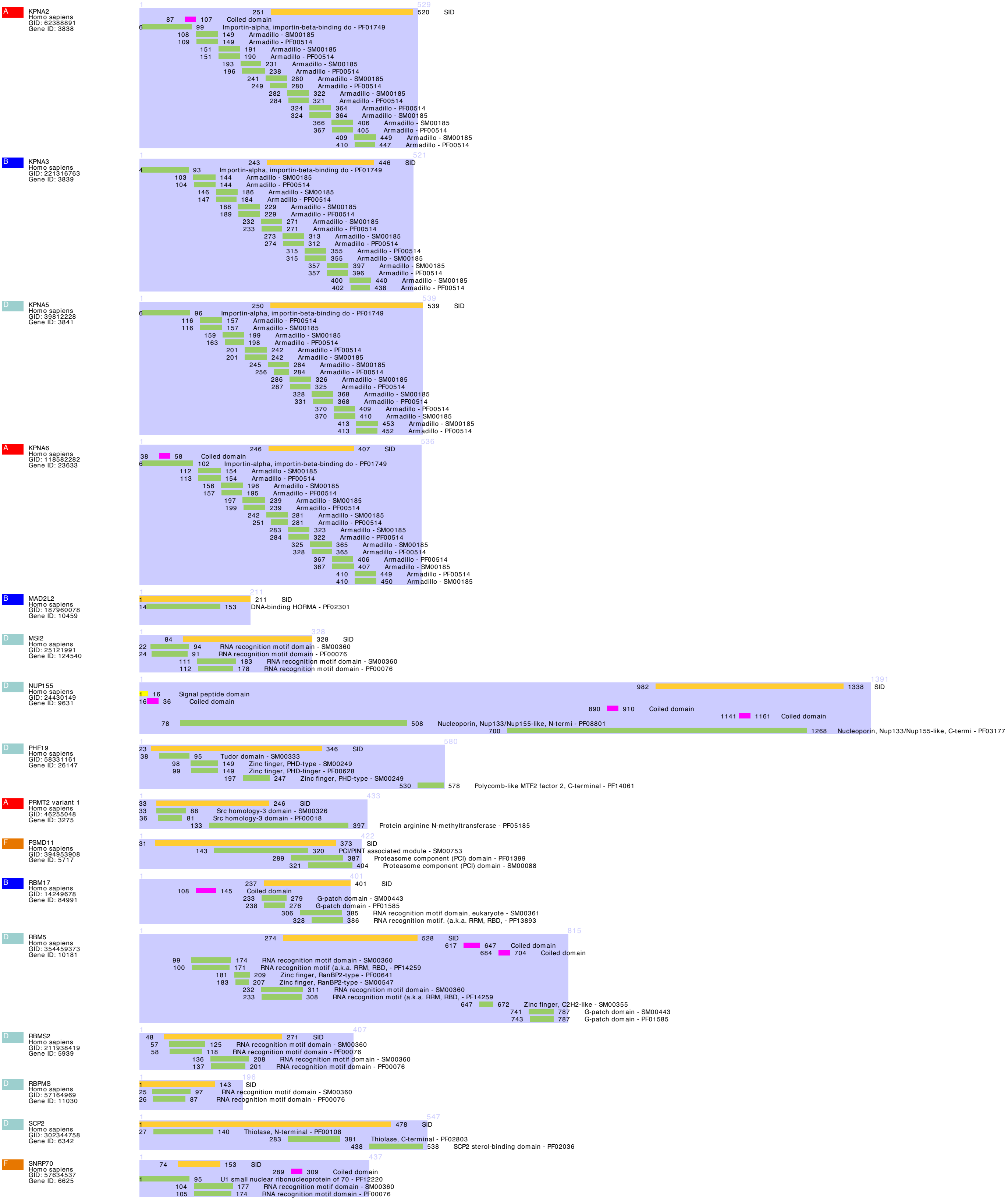

Supplementary Figure S5 - page 3

HBMEC - Human bone marrow endothelial cells

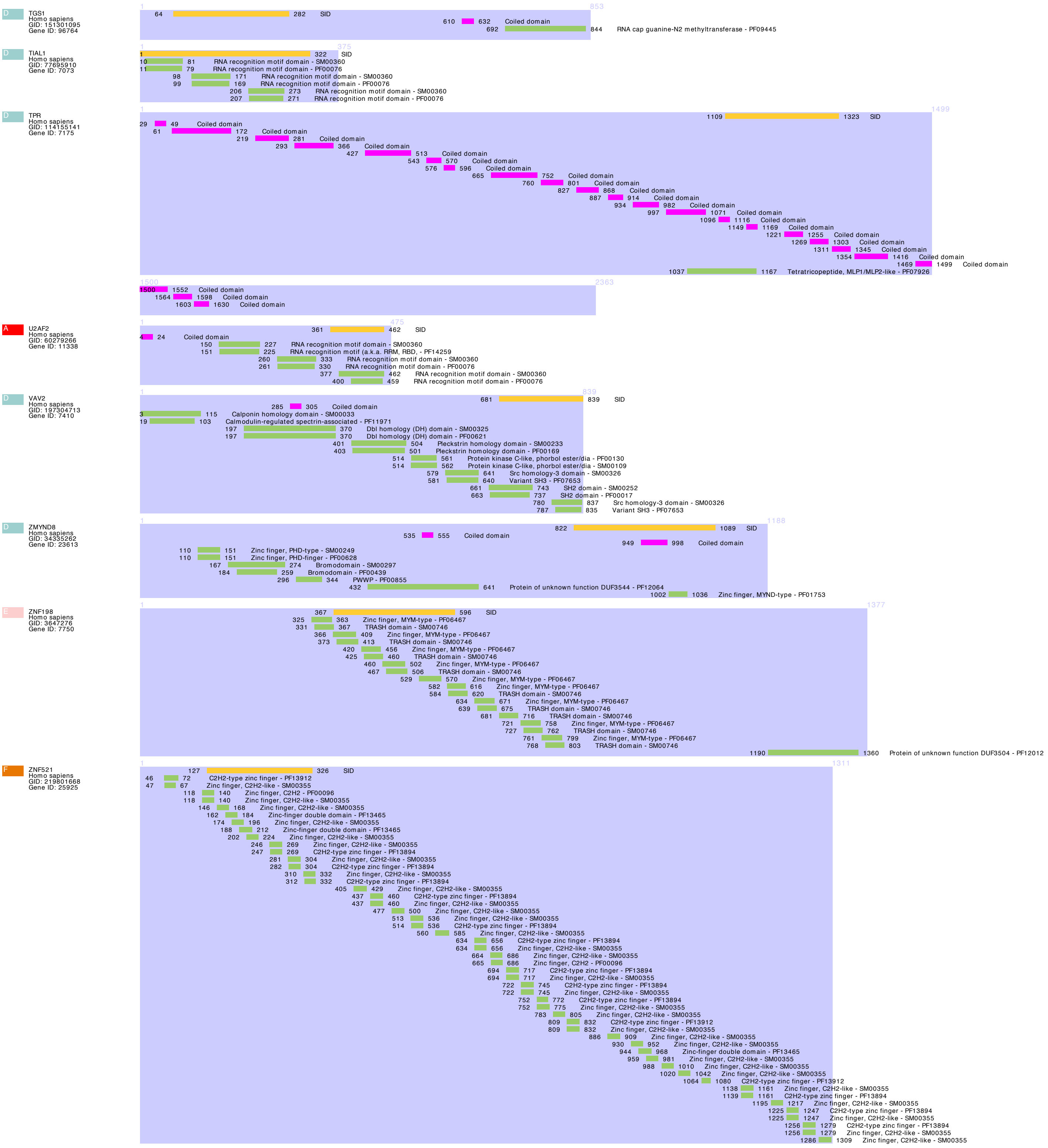

Supplementary Figure S6 - page 1

HTH_RP - Human thymocytes, random-primed

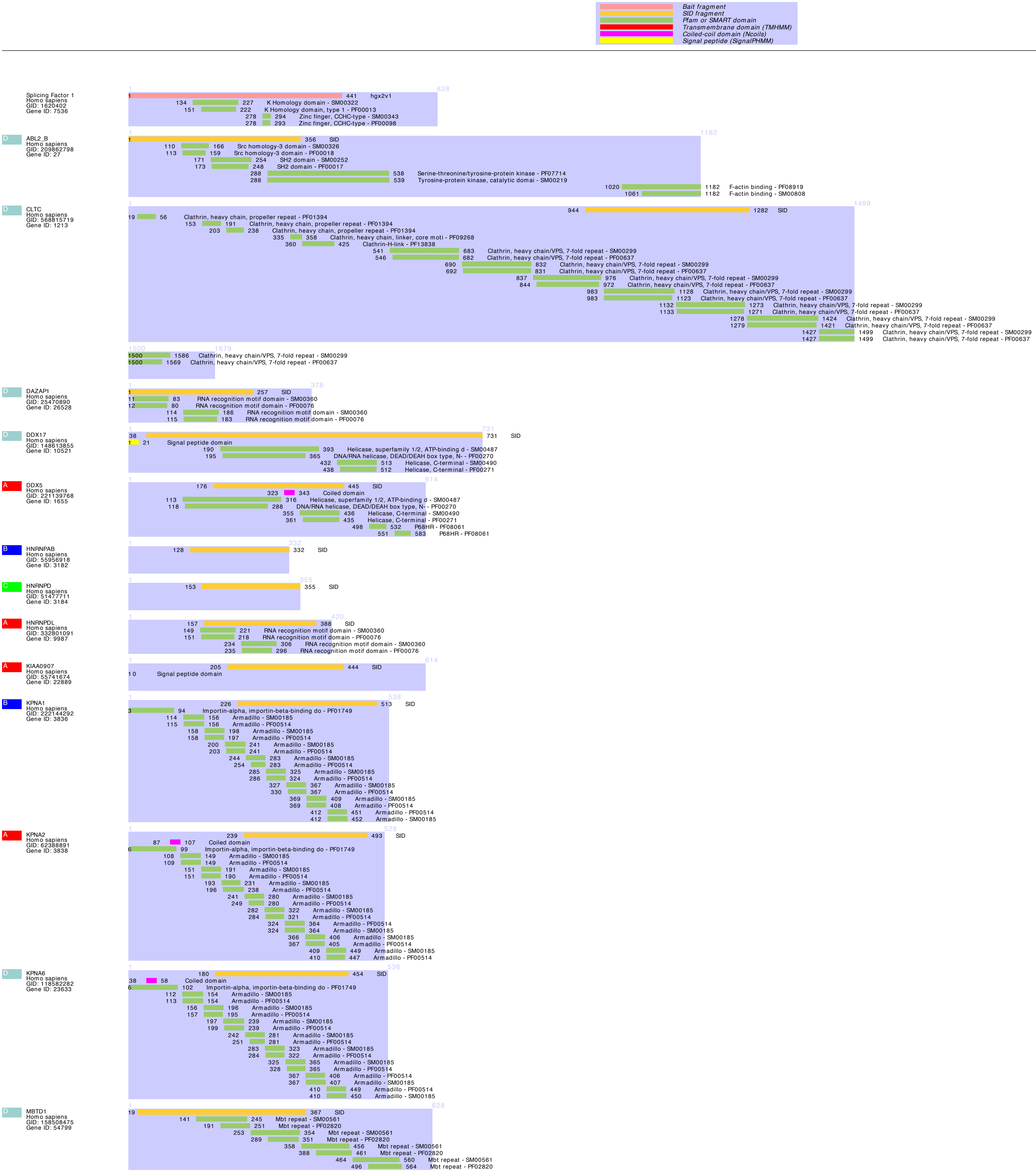

Supplementary Figure S6 - page 2

HTH_RP - Human tyhmocytes

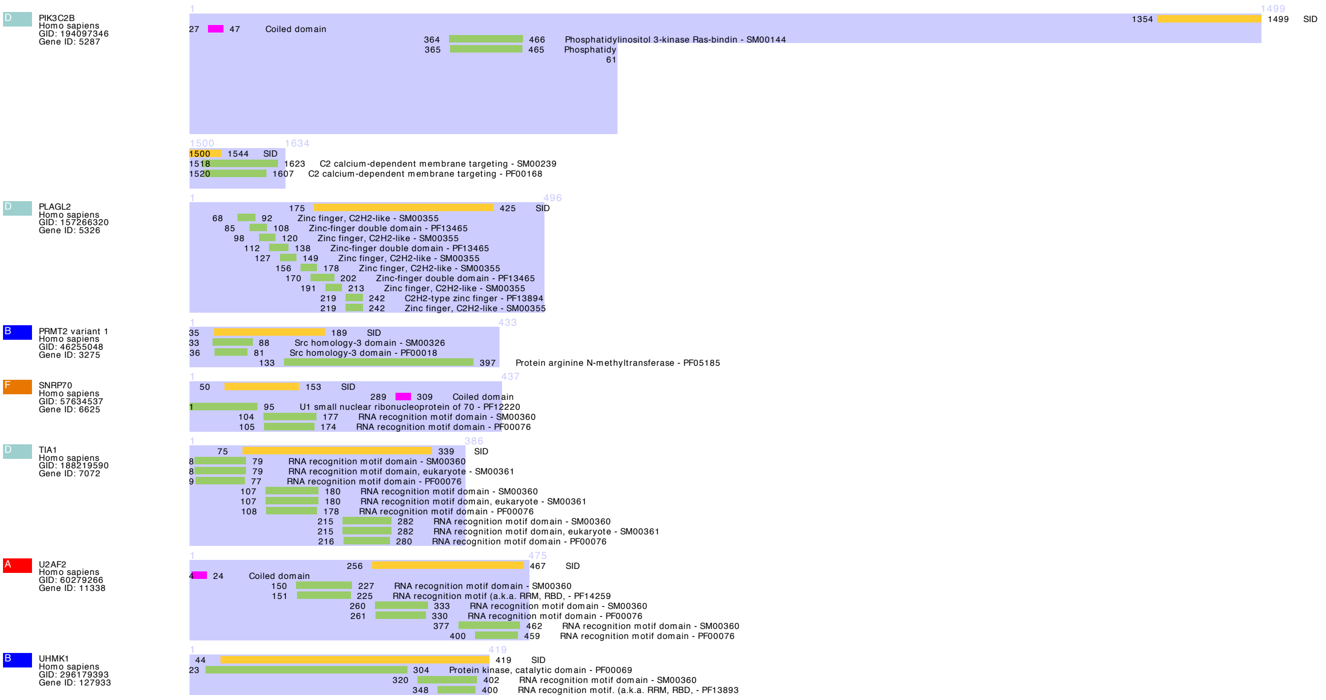

Supplementary Figure S7 - page 1

HTH_dT - Human thymocytes, oligo-dT-primed

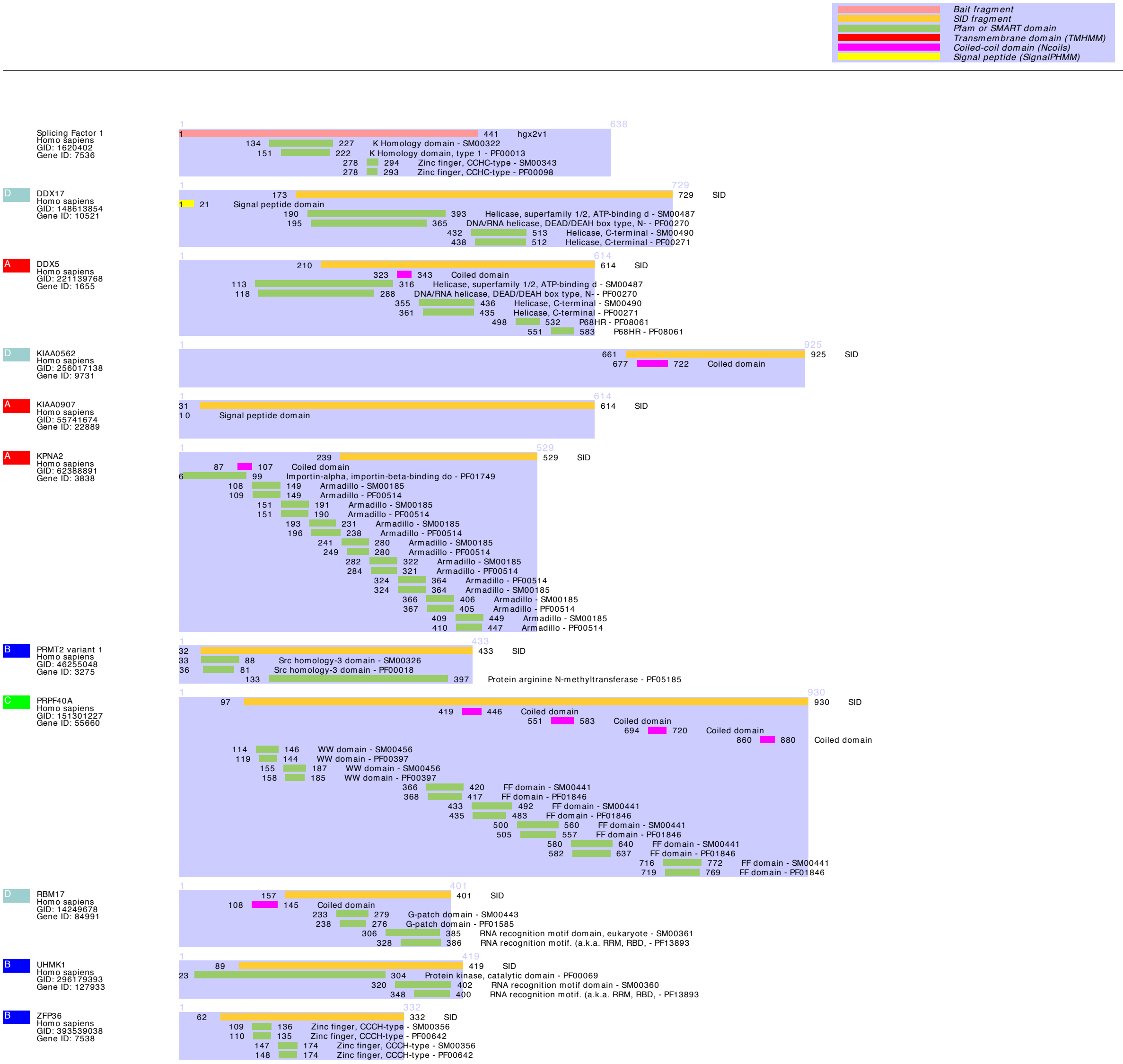

Supplementary Figure S8 - page 1

PLA - Human placenta

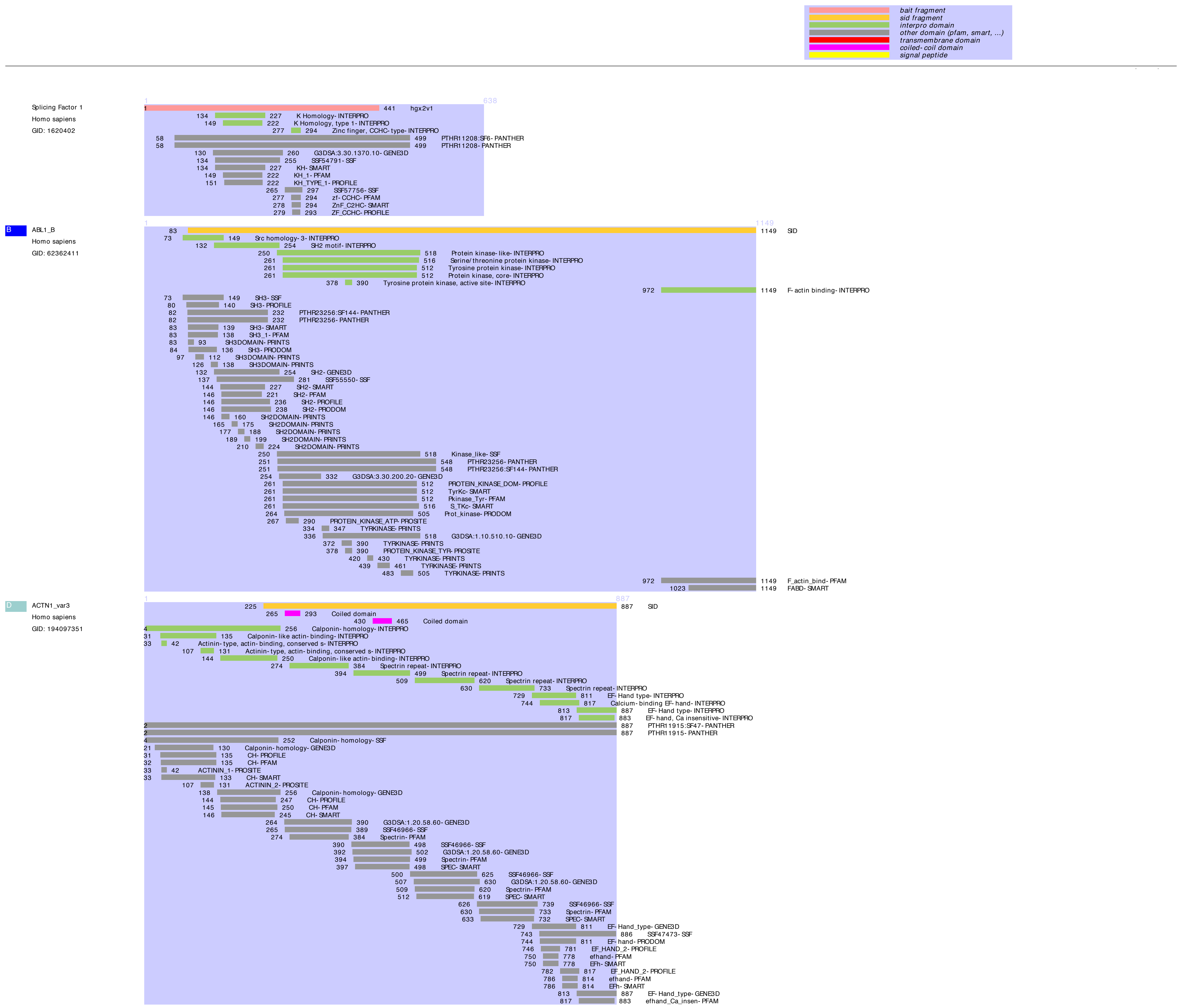

Supplementary Figure S8 - page 2

PLA - Human placenta

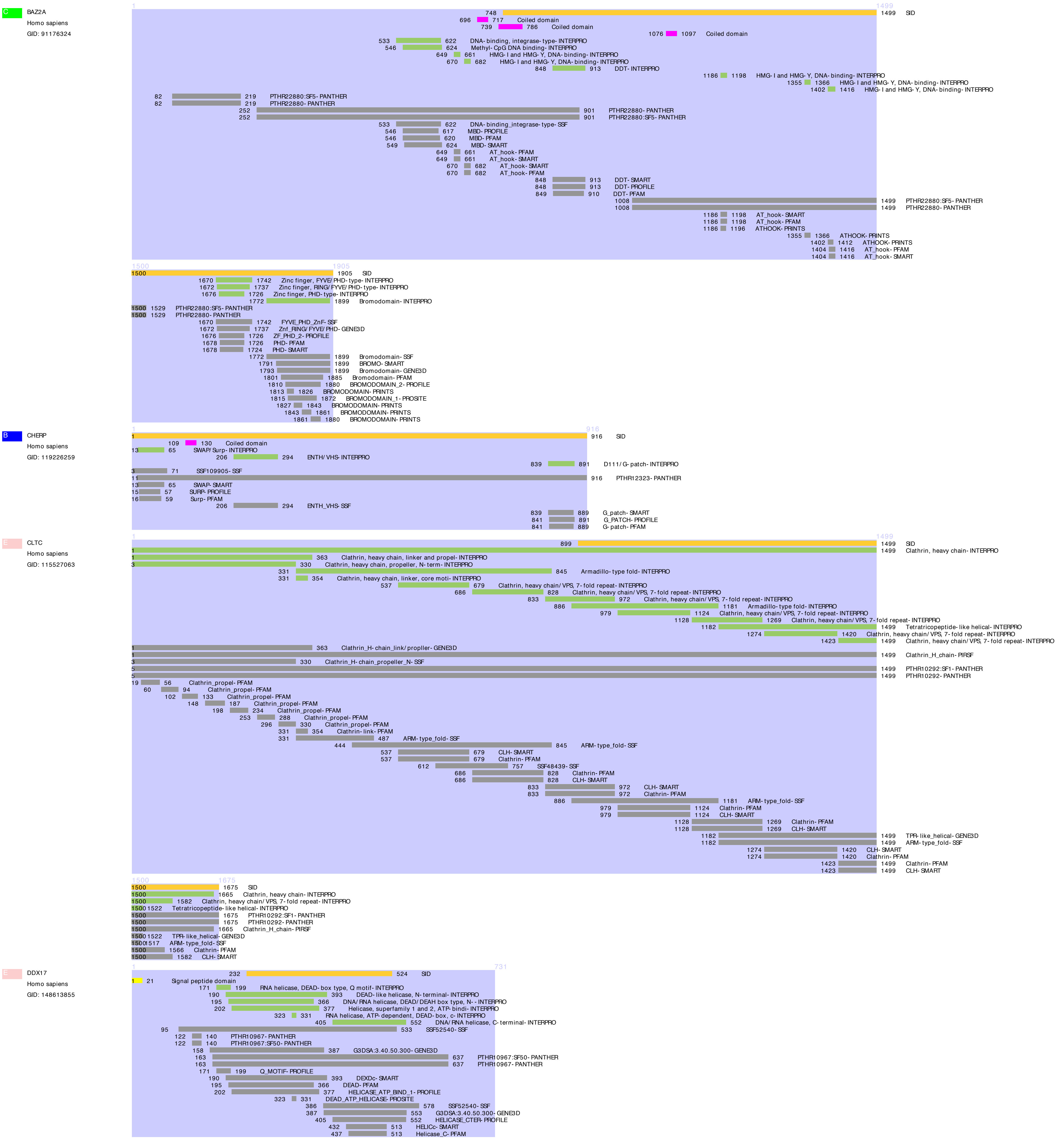

Supplementary Figure S8 - page 3

PLA - Human placenta

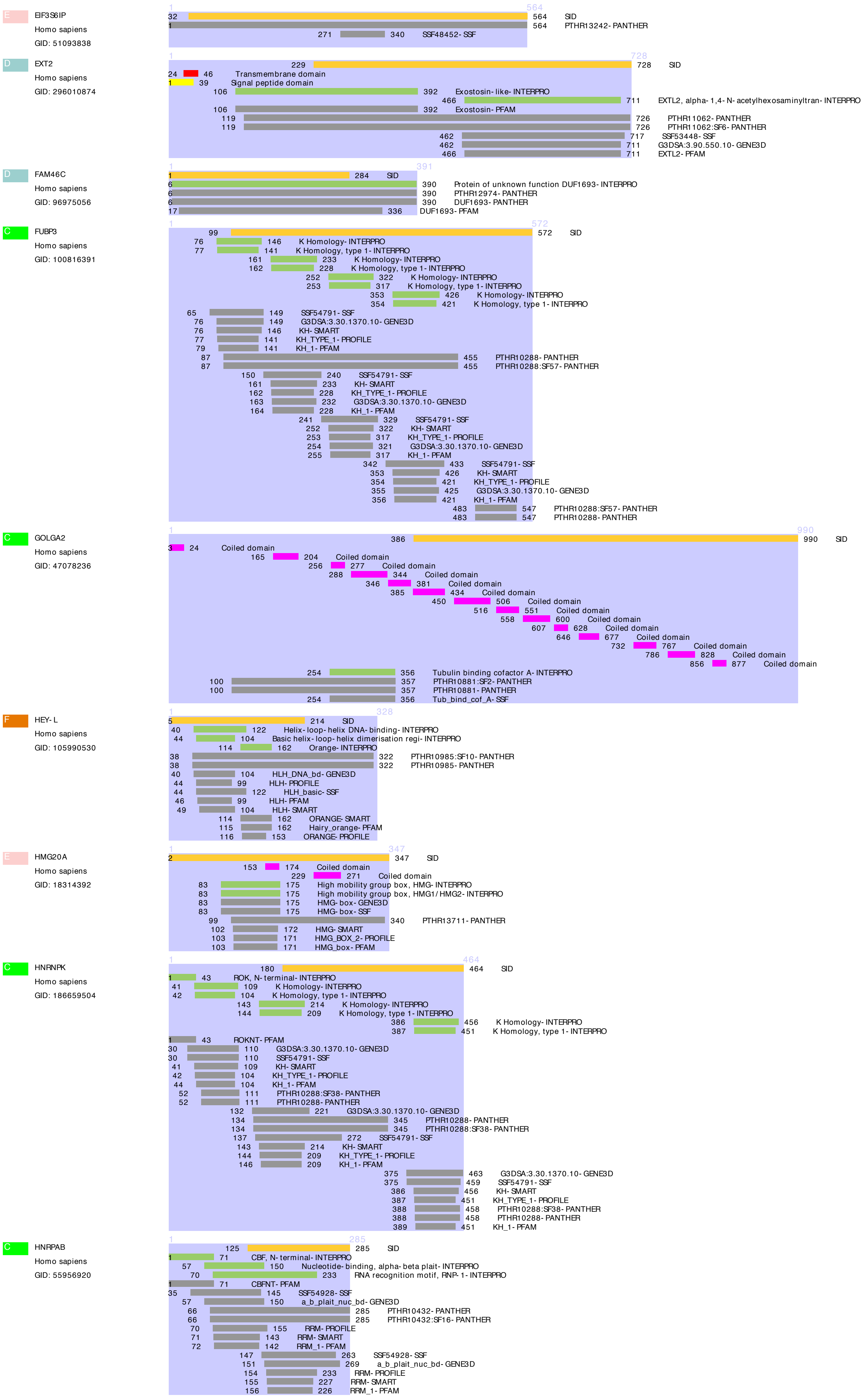

Supplementary Figure S8 - page 4

PLA - Human placenta

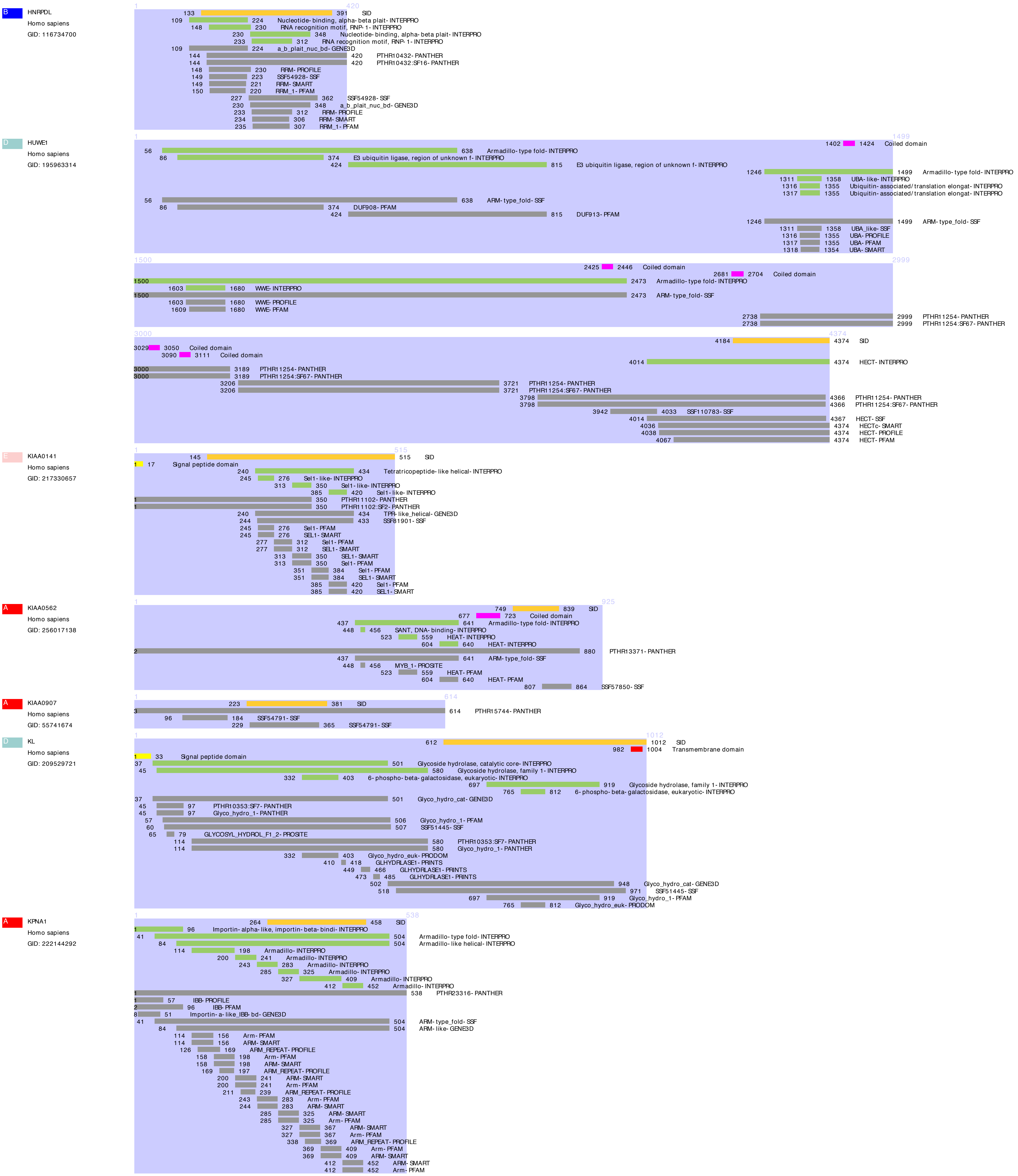

Supplementary Figure S8 - page 5

PLA - Human placenta

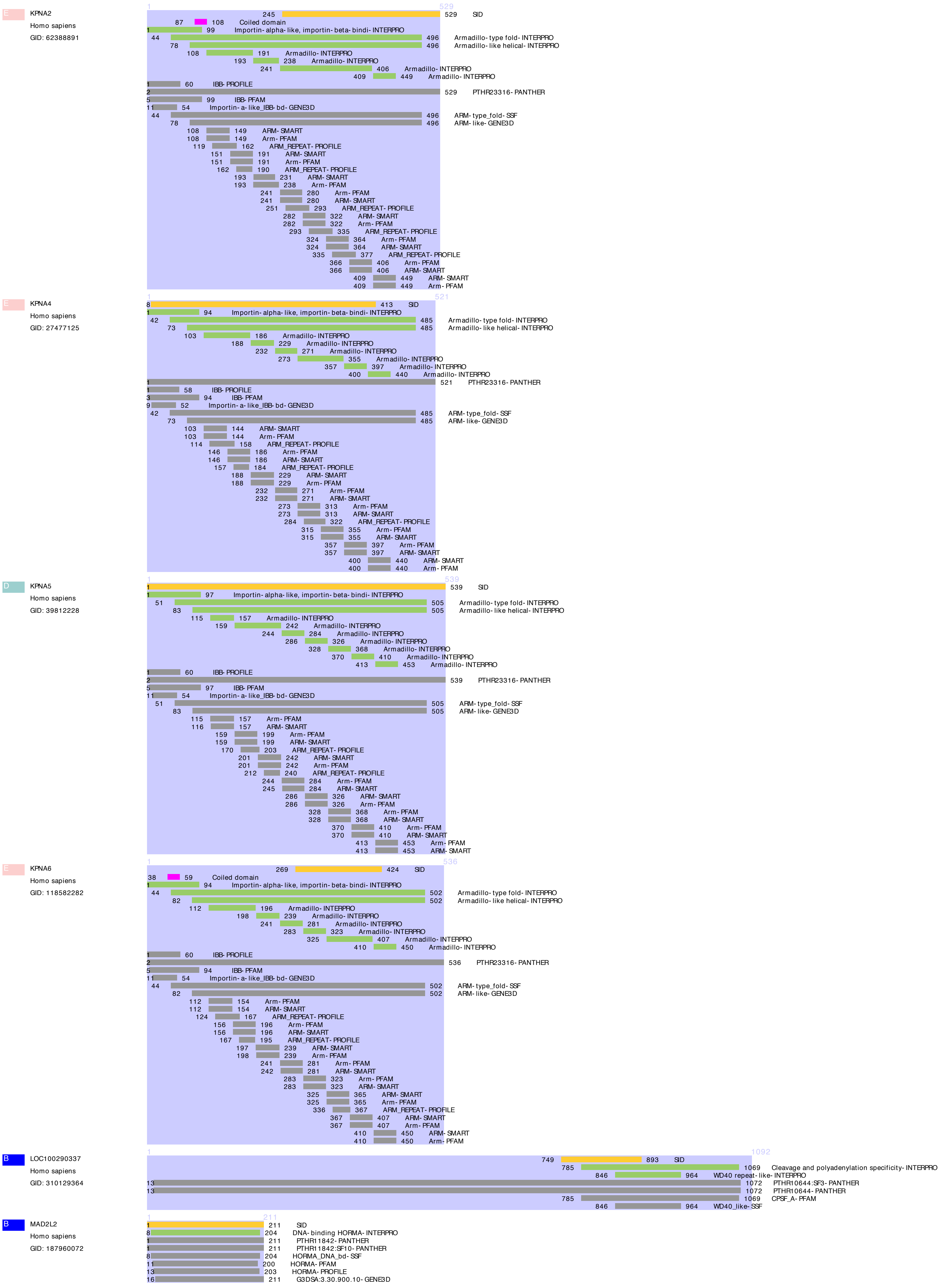

Supplementary Figure S8 - page 6

PLA - Human placenta

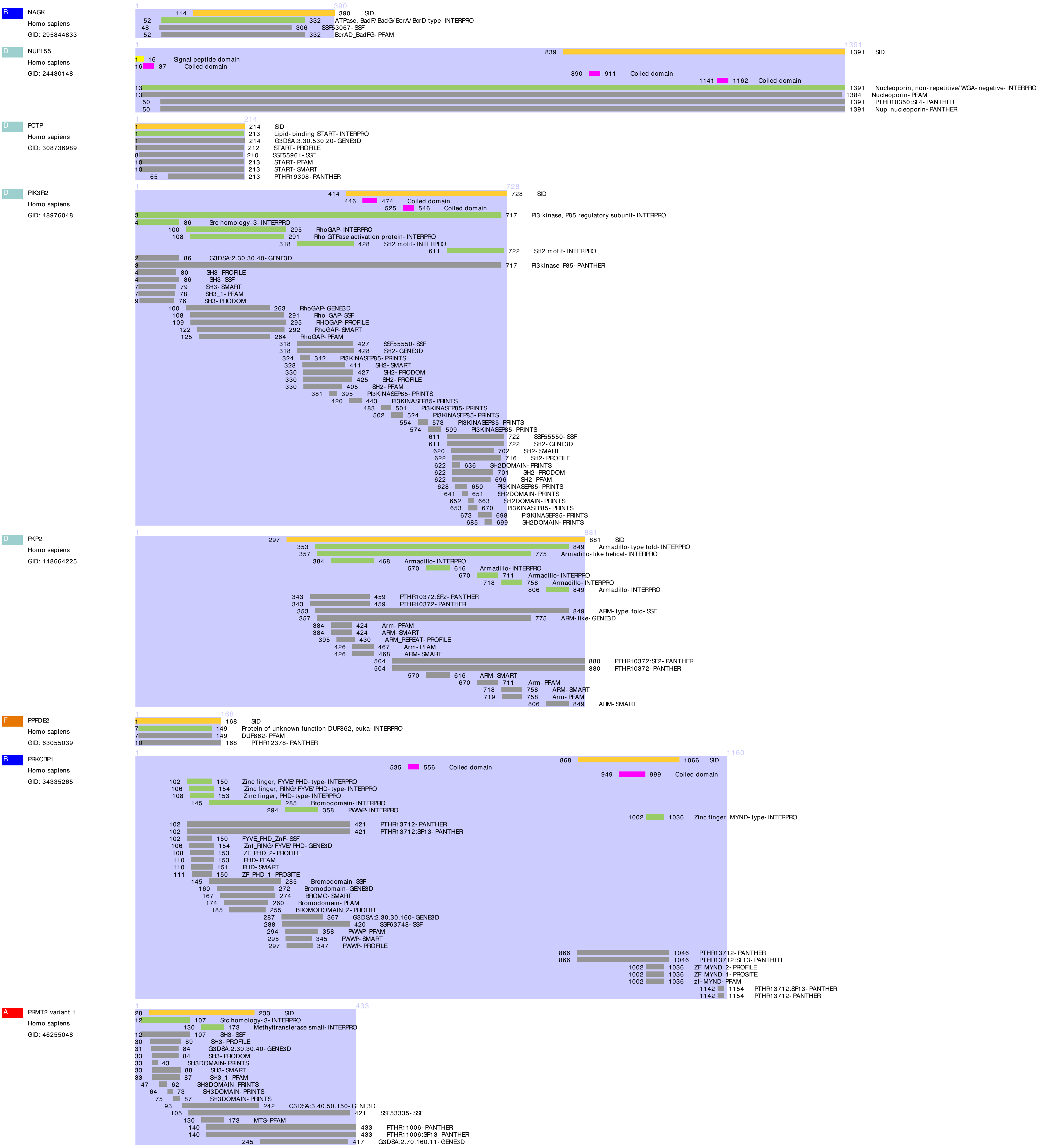

Supplementary Figure S8 - page 7

PLA - Human placenta

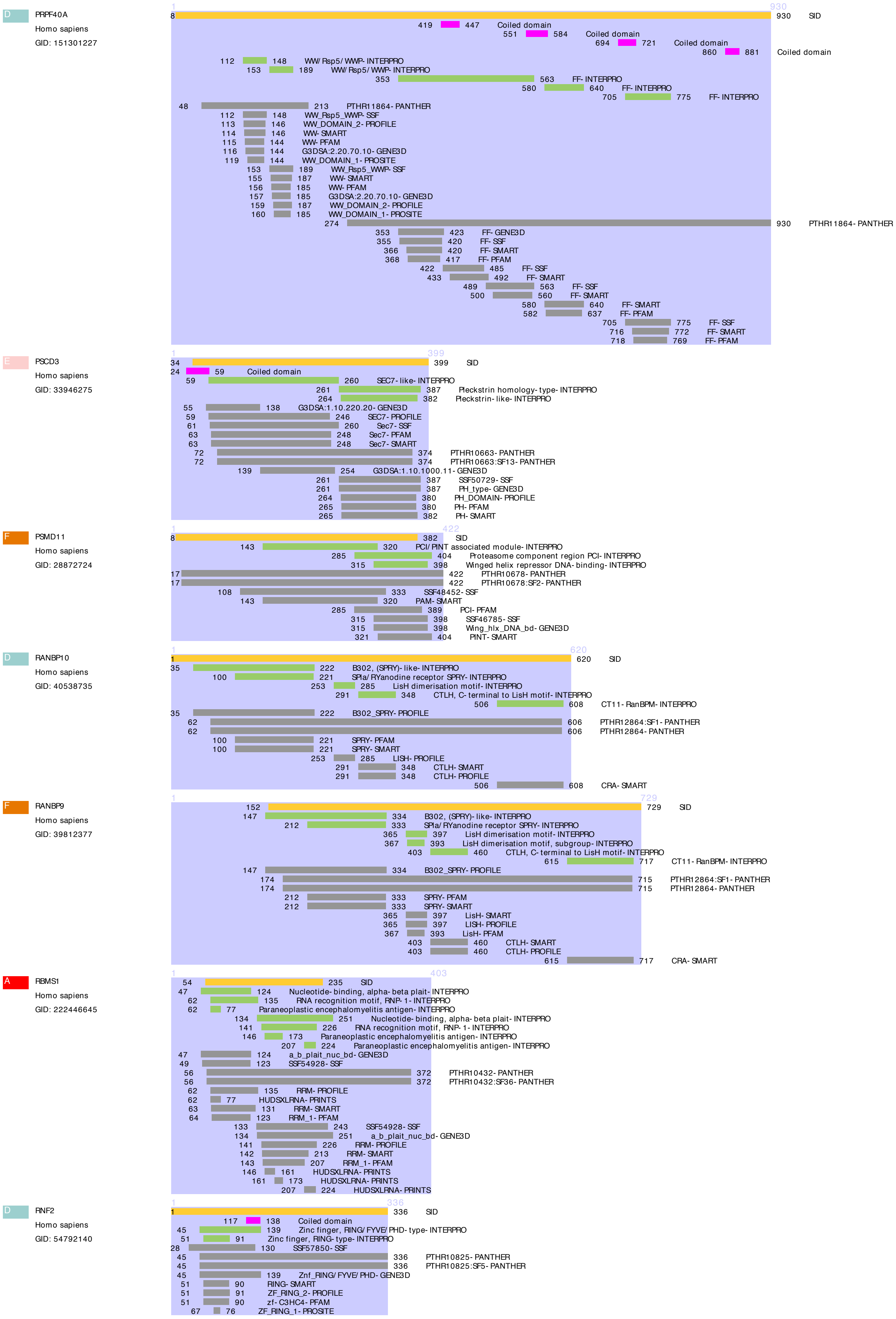

Supplementary Figure S8 - page 8

PLA - Human placenta

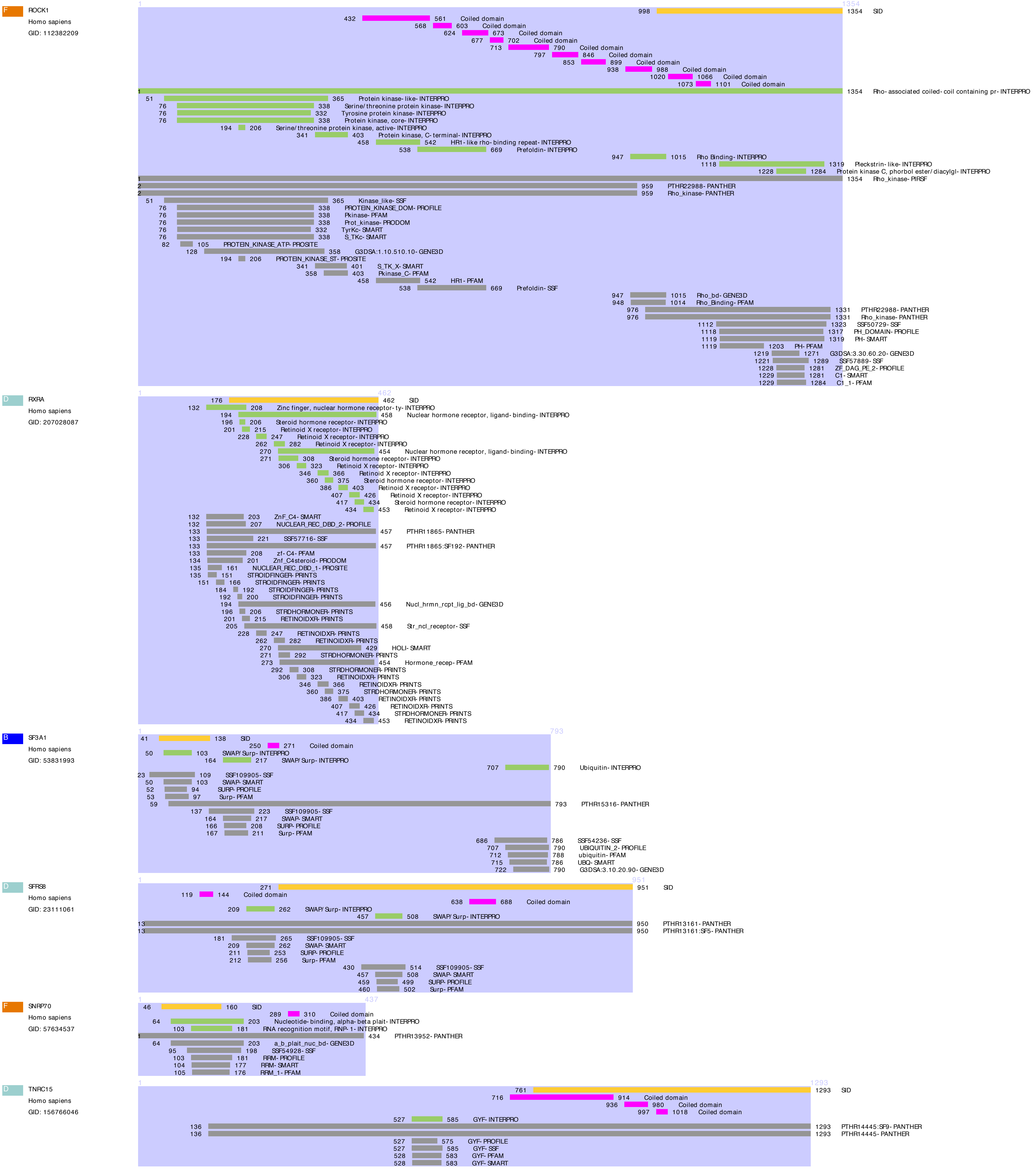

Supplementary Figure S8 - page 9

PLA - Human placenta

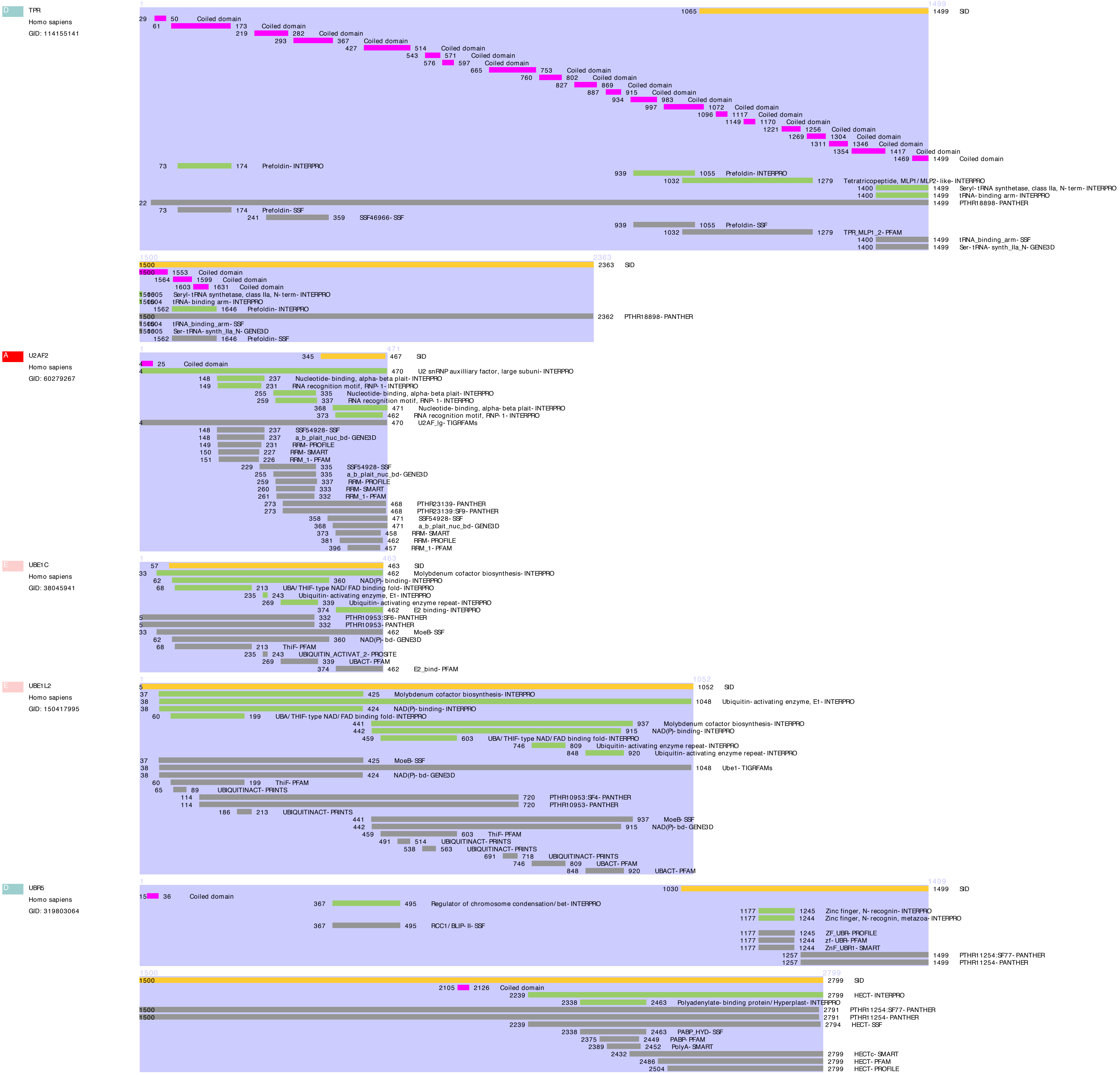

Supplementary Figure S8 - page 10

PLA - Human placenta

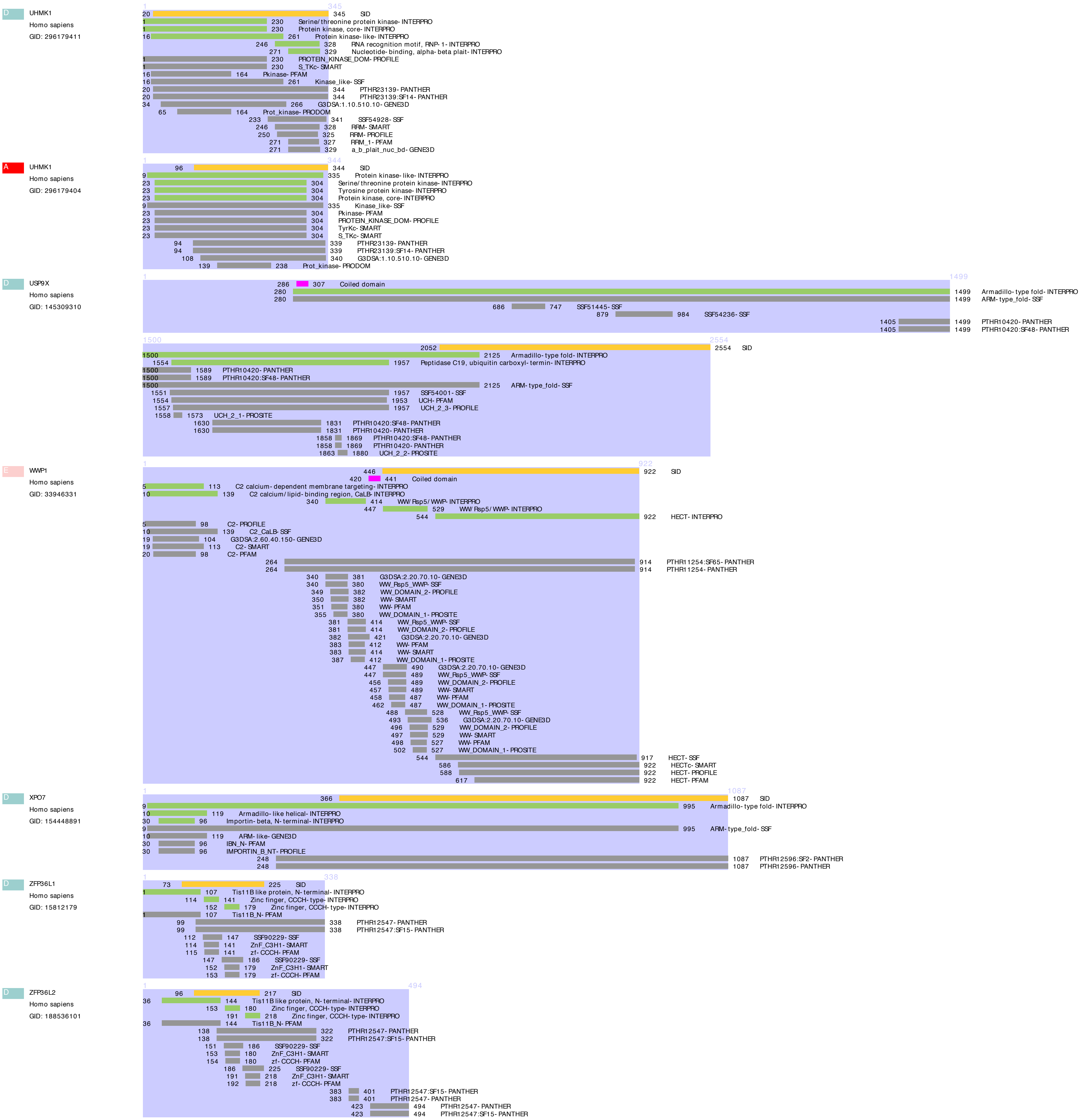

Supplementary Figure S8 - page 11

PLA - Human placenta

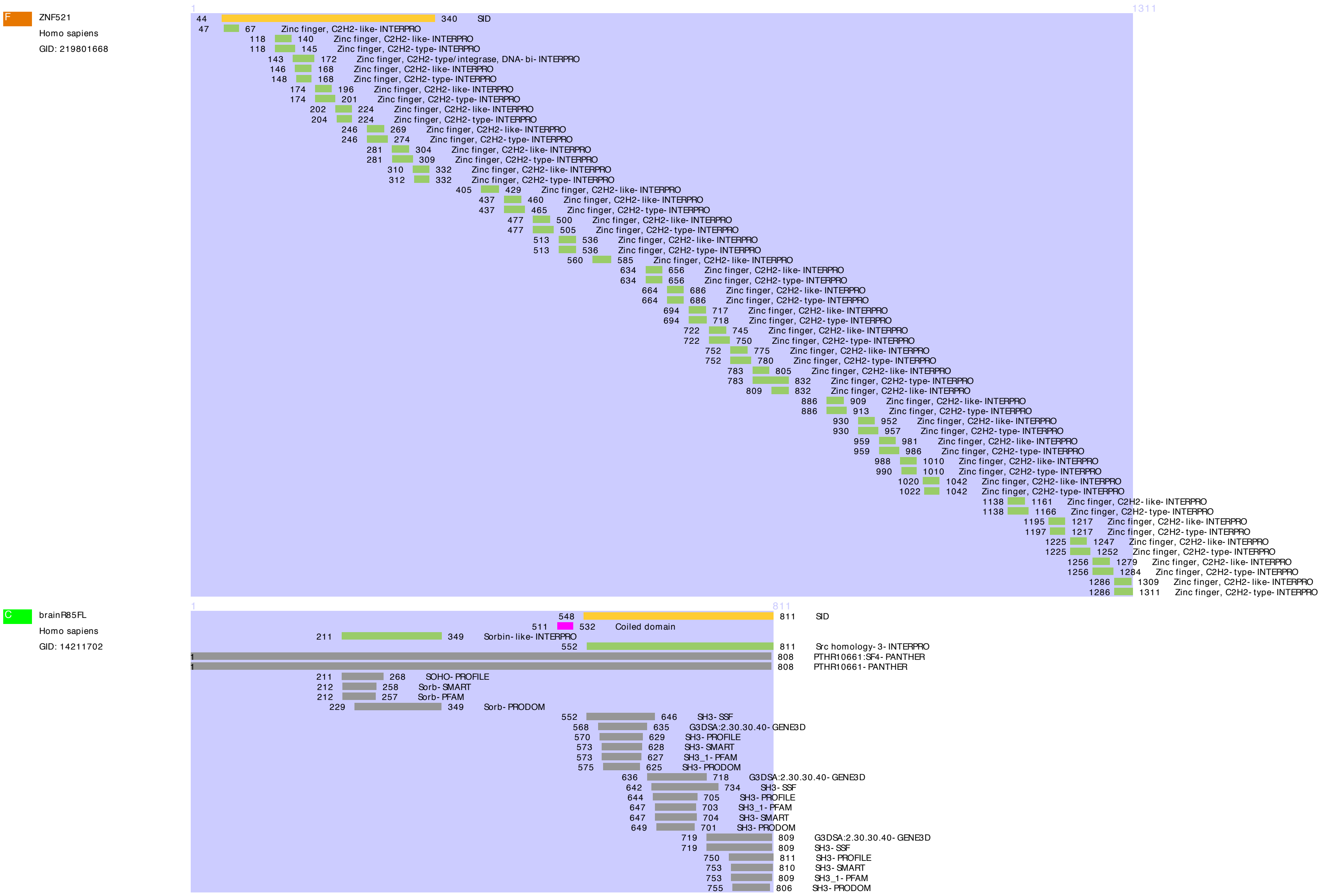

Supplementary Figure S9 - page 1

MANE - Mouse adult neurosphere cells

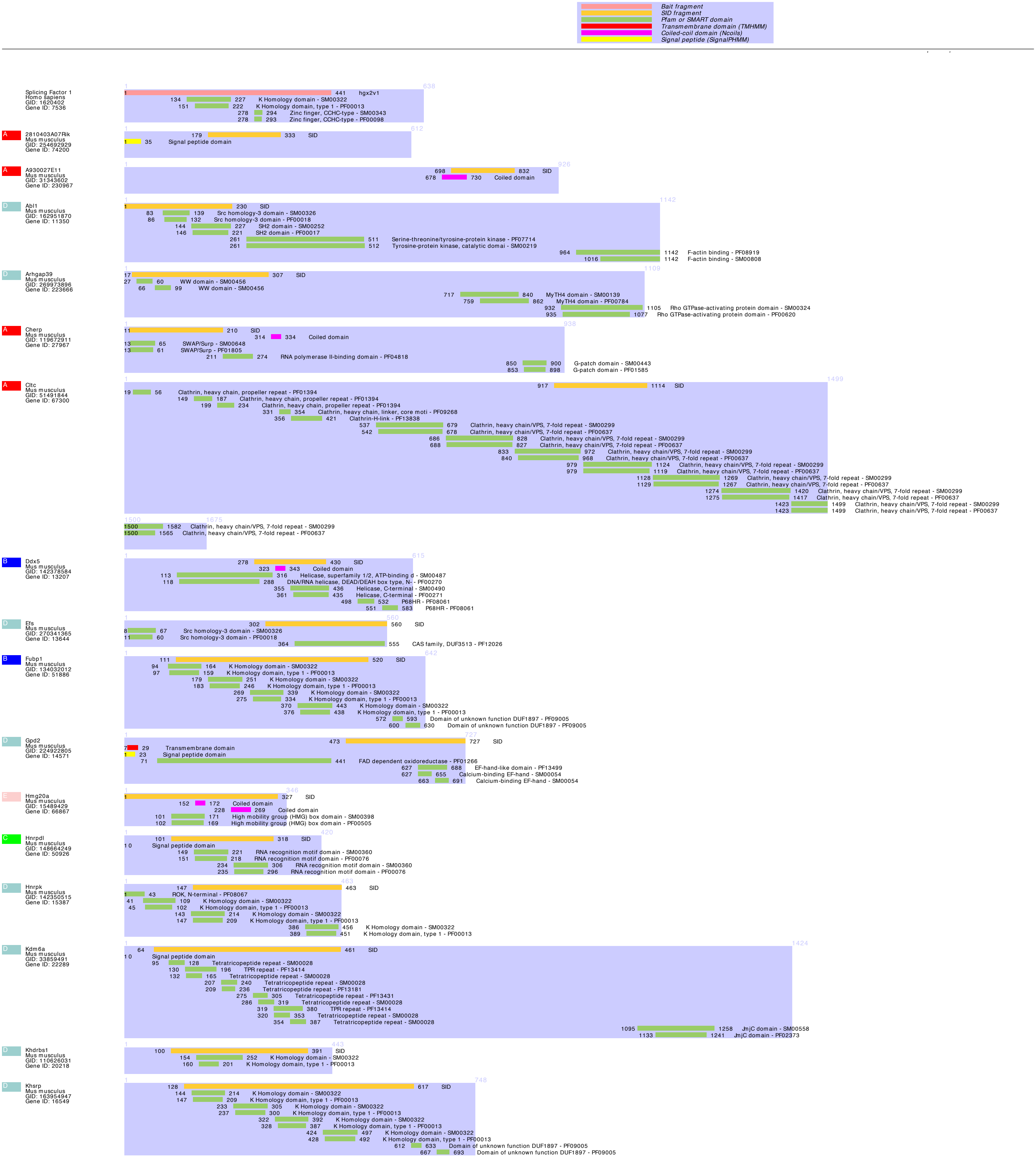

Supplementary Figure S9 - page 2

MANE - Mouse adult neurosphere cells

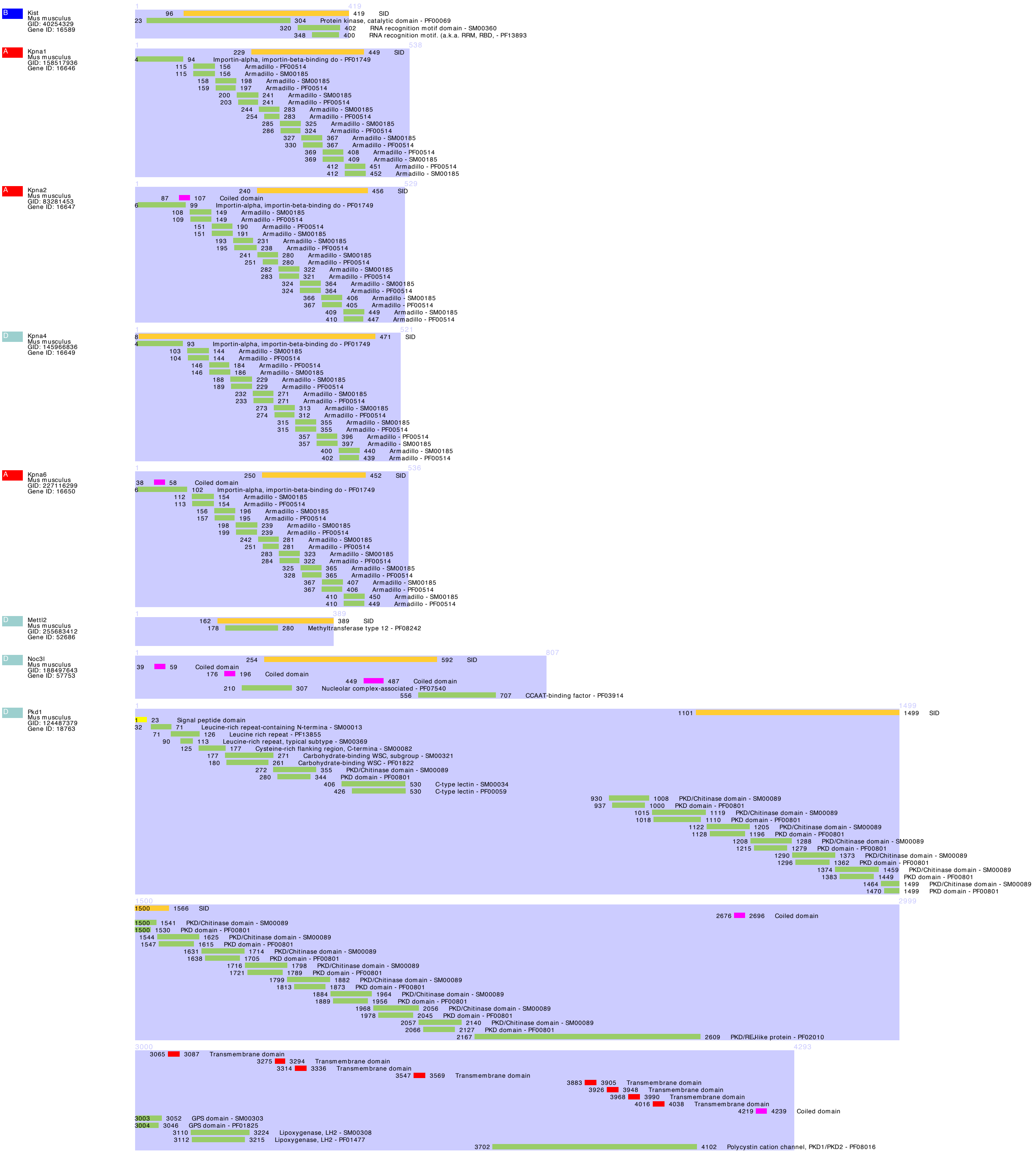

Supplementary Figure S9 - page 3

MANE - Mouse adult neurosphere cells

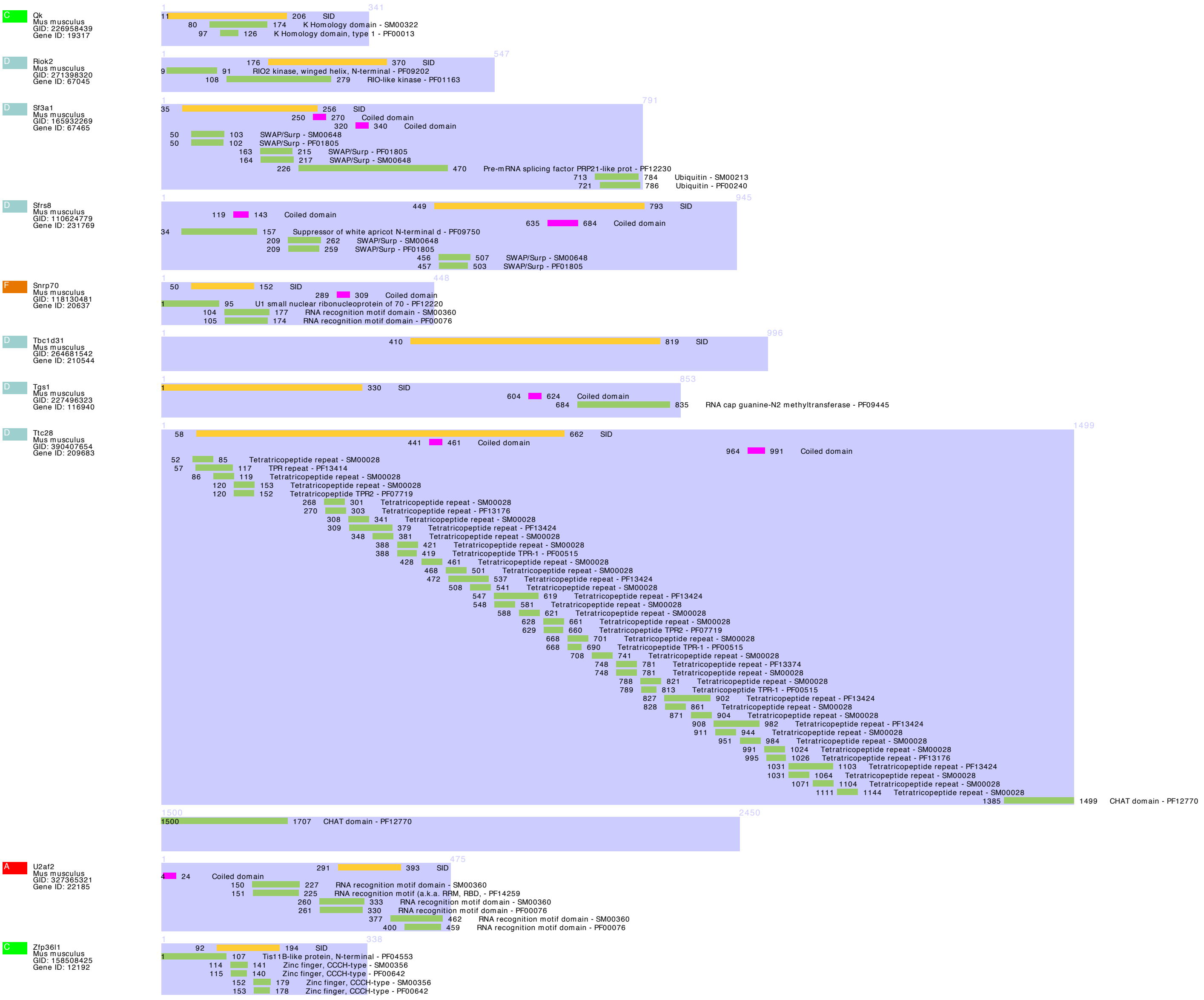

Supplementary Figure S9 - page 4

MANE - Mouse adult neurosphere cells

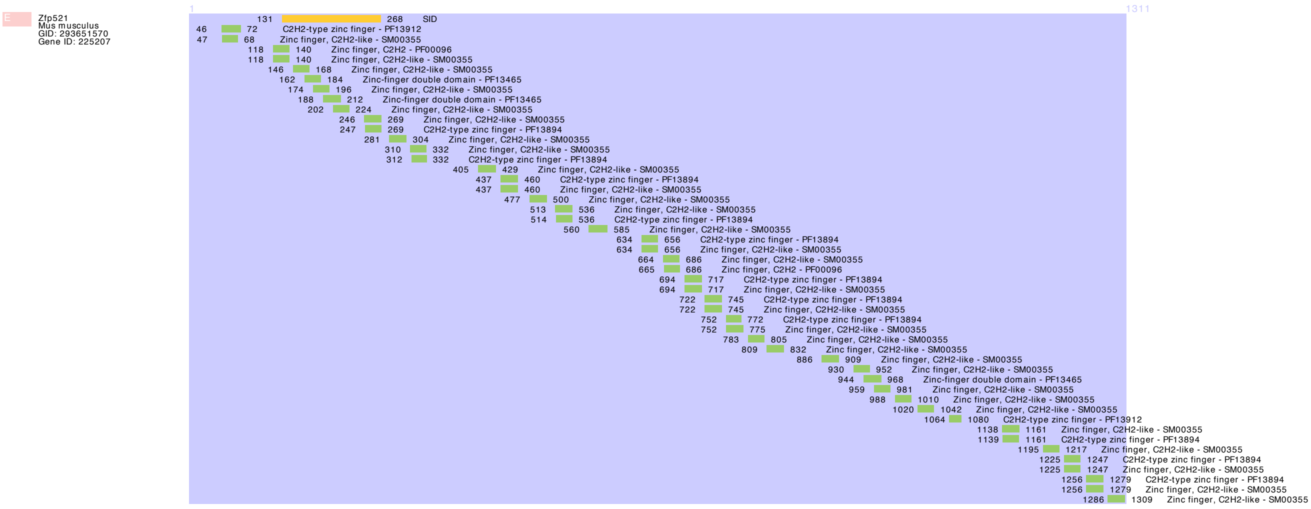

Supplementary Figure S10 - page 1

MKI - Total mouse kidney

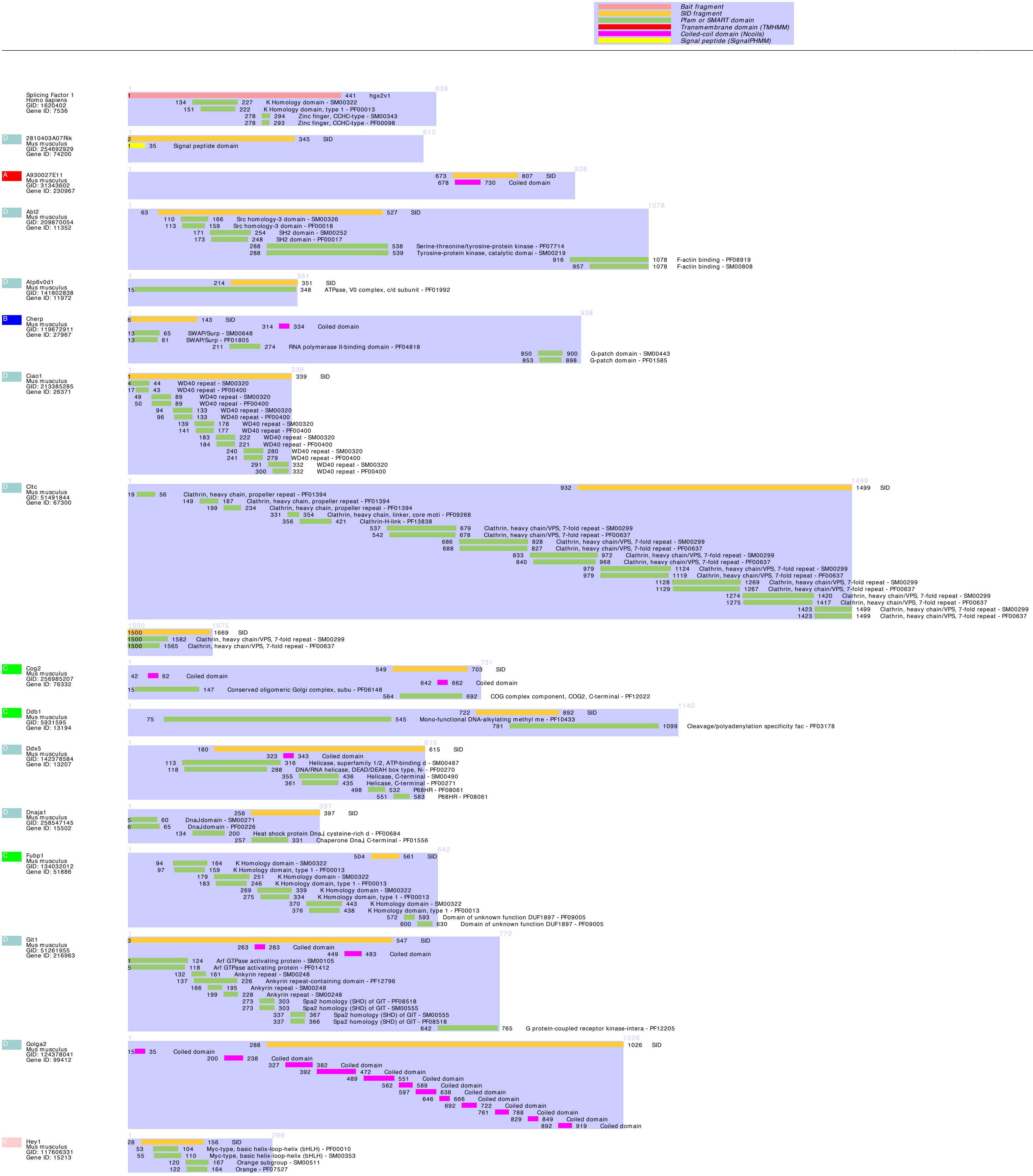

Supplementary Figure S10 - page 2

MKI - Total mouse kidney

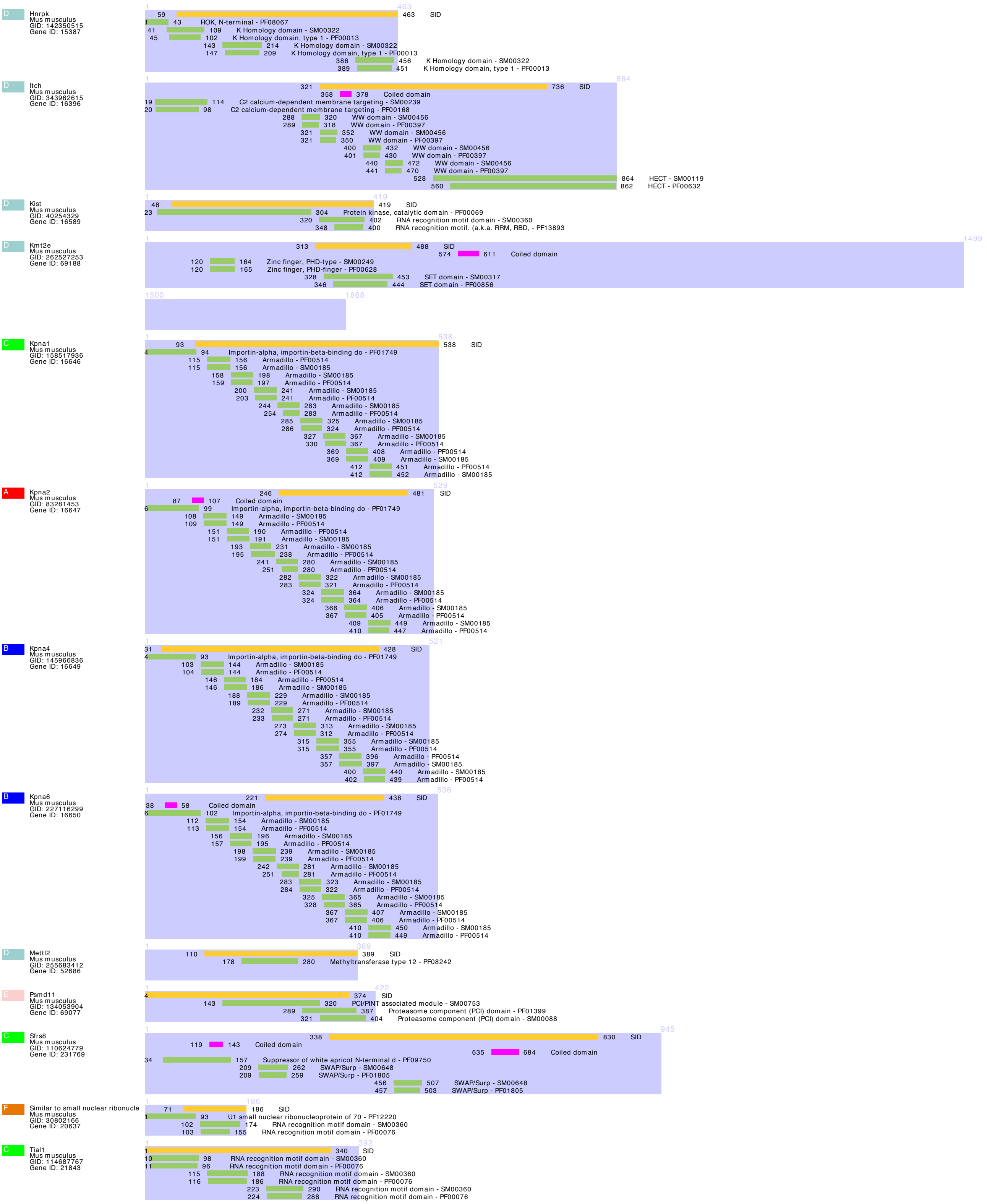

Supplementary Figure S10 - page 3

MKI - Total mouse kidney

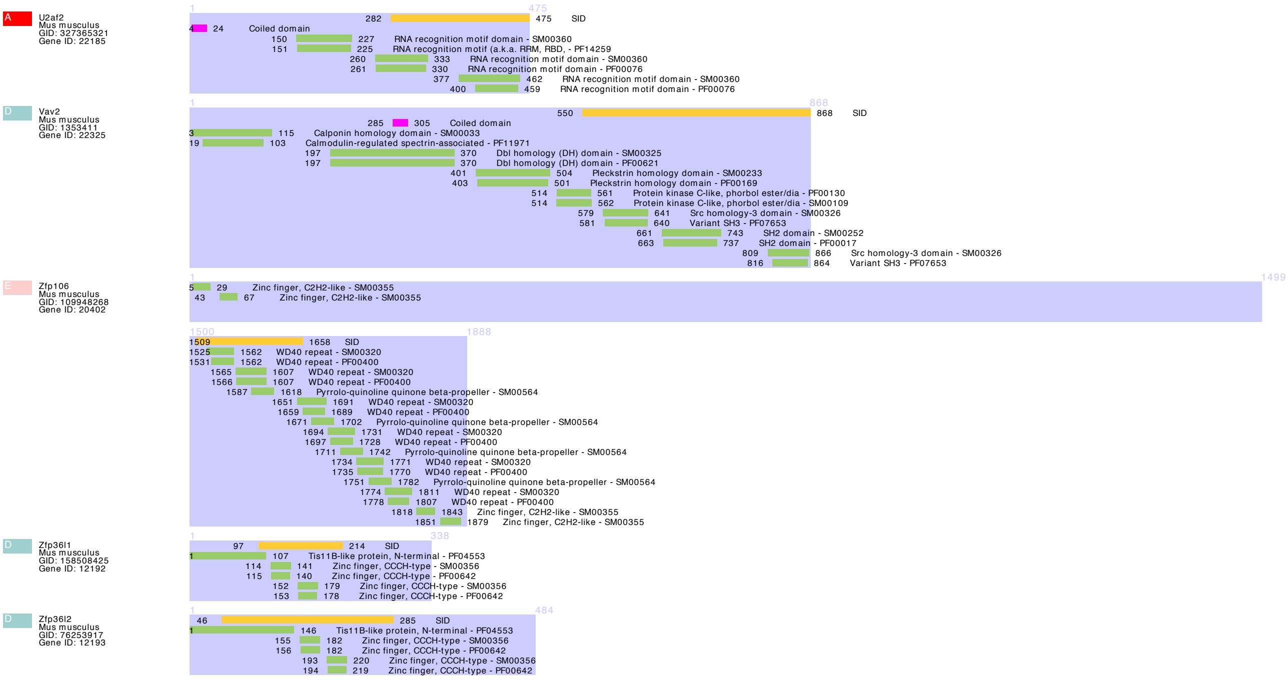

Supplementary Figure S11 - page 1

MPC - Mouse pancreatic cells

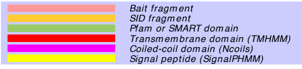

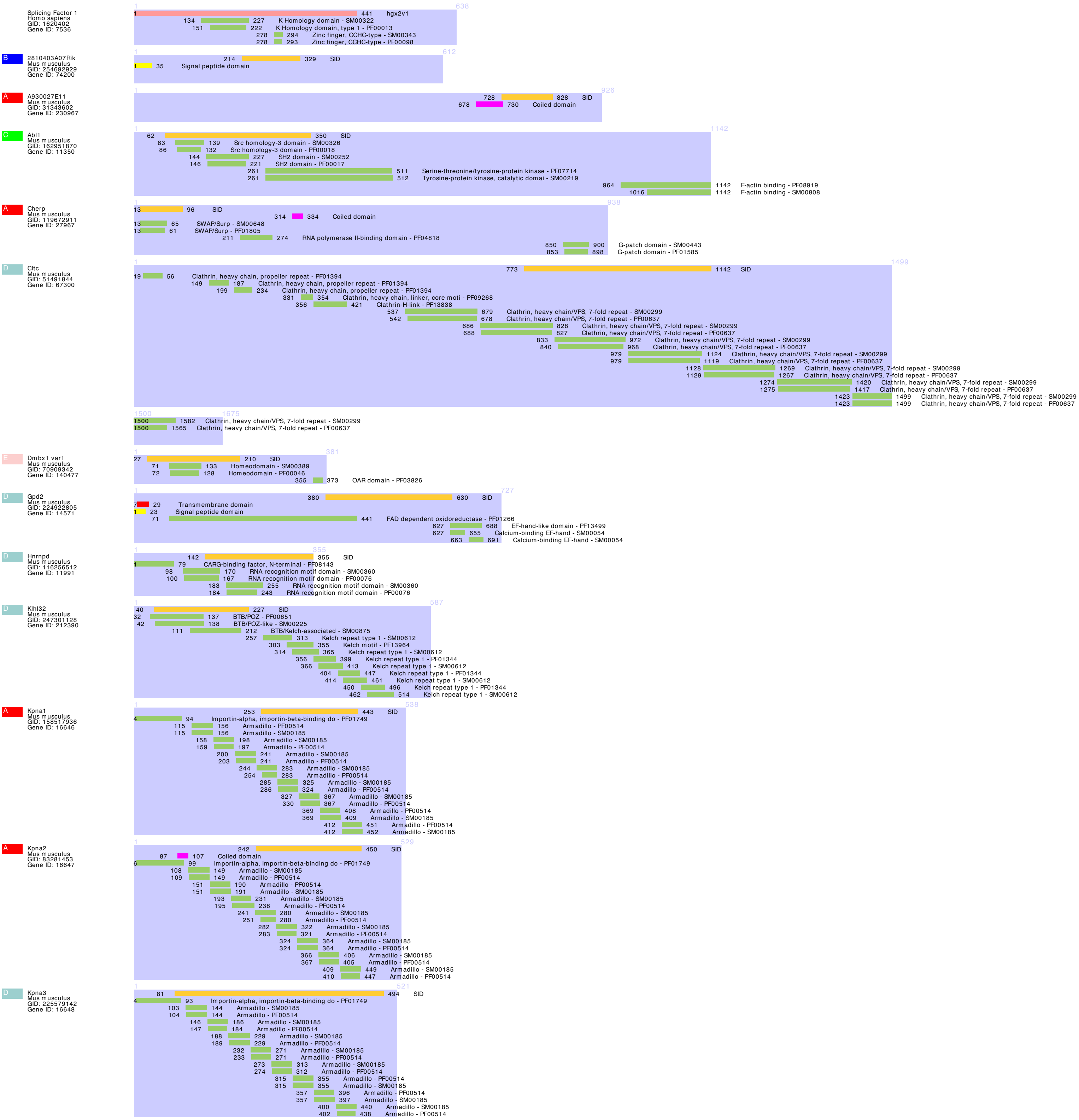

Supplementary Figure S11 - page 2

MPC - Mouse pancreatic cells

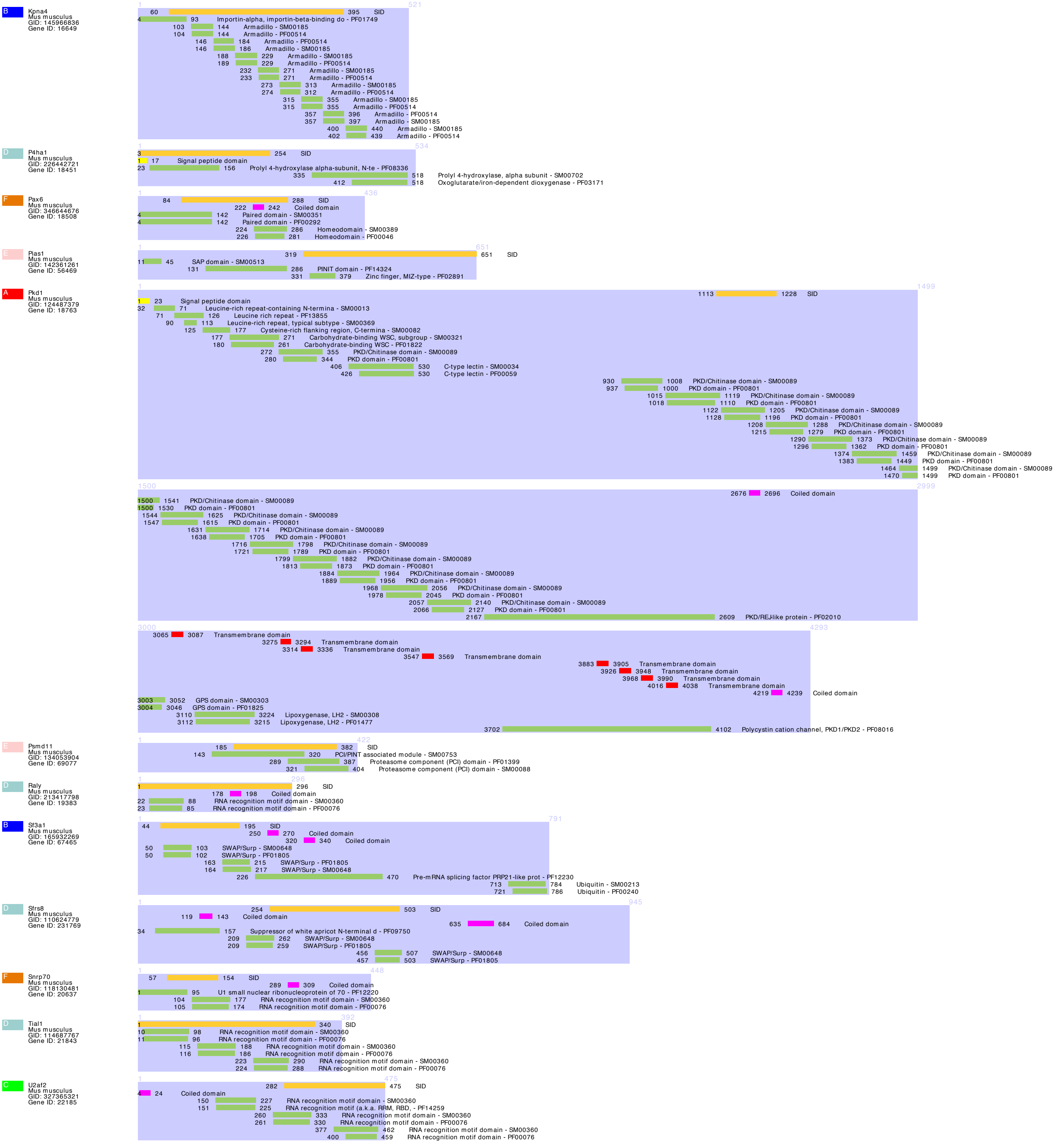

Supplementary Figure S11 - page 3

MPC - Mouse pancreatic cells

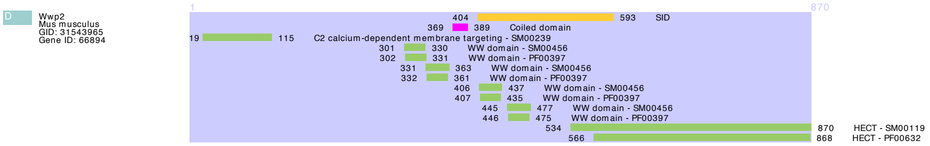

Supplement: SUPPLEMENTARY DATA [file supp_gkv952_nar-00201-a-2015-File009.pdf]
