# Supplementary material for: Mammalian splicing factor SF1 interacts with SURP domains of U2 snRNP-associated proteins
Source: Nucleic Acids Res. 2015 Sep 29;43(21):10456–73. doi: 10.1093/nar/gkv952 (PMC4666396; doi:10.1093/nar/gkv952)
Supplement: SUPPLEMENTARY DATA [file supp_gkv952_nar-00201-a-2015-File010.pdf]

Supplementary Table S1. Proteins immunoprecipitated with anti-SF1 antibodies from HeLa cell nuclear extracts with less than five spectrum counts and potential contaminants not included in Table 1.

				SF1		IgG	
Name[1]	Symbol[2]	Gene ID[3]	Group[4]	- RNase	+ RNase	- RNase	+ RNase
**Spliceosome-associated proteins according to Hegele et al. (49)**							
SF3a66	SF3A2	8175	U2	4	4	0	0
FLJ10839	CCAR1	55749	A	0	1	0	0
FLJ10154	ARGLU1	55082	SR	3	1	0	0
40K	SNRNP40	9410	U5	4	0	0	0
hSAD1	USP39	10713	U4/U6.U5	3	4	0	0
LSm6	LSM6	11157	LSm	3	0	0	0
LSm2	LSM2	57819	LSm	3	0	0	0
LSm4	LSM4	25804	LSm	1	0	0	0
PRL1	PLRG1	5356	Prp19	3	2	0	0
SKIP	SNW1	22938	Prp19 rel	3	1	0	0
THRAP3	THRAP3	9967	B	1	0	0	0
Aly	ALYREF	10189	EJC/TREX	3	1	0	0
Magoh	MAGOH	4116	EJC/TREX	4	0	0	0
YB-1	YBX1	4904	mRNA	2	1	0	0
**Proteins not known to be associated with spliceosomes and potential contaminants**							
	CP	1356		10	8	0	1
	RPS13	6207		5	7	0	0
	NELFE	7936		0	4	0	0
	CPSF7	79869		3	3	0	0
	GEMIN5	25929		3	3	0	0
	P3H1	64175		3	3	0	0
	DNMT1	1786		1	3	0	0
	LUC7L2	51631		0	3	0	0
	PHGDH	26227		0	3	0	0
	TNS1	7145		0	3	0	0
	FGB	2244		123	2	0	0
	RPS10	6204		4	2	0	0
	HIST1H4A	8359		3	2	0	0
	TROVE2	6738		3	2	0	0
	RPLP0	6175		2	2	0	0
	EEA1	8411		1	2	0	0
	ATM	472		0	2	0	0
	DNAJC7	7266		0	2	0	0
	GGCT	79017		0	2	0	0
	RPS3A	6189		4	1	0	0
	TGM2	7052		3	1	0	0
	FAM120A	23196		2	1	0	0
	RCC2	55920		2	1	0	0
	THOC6	79228		2	1	0	0
	ACACA	31		1	1	0	0
	CCAR2	57805		1	1	0	0
	FSCN1	6624		1	1	0	0

Name[1]	Symbol[2]	Gene ID[3]	Group[4]	SF1		IgG	
				- RNase	+ RNase	- RNase	+ RNase
	GRB2	2885		1	1	0	0
	IPO5	3843		1	1	0	0
	SMC4	10051		1	1	0	0
	TFCP2	7024		1	1	0	0
	TPT1	7178		1	1	0	0
	ABCF1	23		0	1	0	0
	ANXA5	308		0	1	0	0
	ATP5A1	498		0	1	0	0
	BTF3	689		0	1	0	0
	CSTF2	1478		0	1	0	0
	DARS	1615		0	1	0	0
	FANCI	55215		0	1	0	0
	FKBP4	2288		0	1	0	0
	HLA-B	3106		0	1	0	0
	IPO7	10527		0	1	0	0
	LRPPRC	10128		0	1	0	0
	RAB3GAP2	25782		0	1	0	0
	SLC3A2	6520		0	1	0	0
	UGGT1	56886		0	1	0	0
	WARS	7453		0	1	0	0
	FGG	2266		103	0	0	0
	KRT13	3860		11	0	0	0
	MDH2	4191		5	0	0	0
	EIF3D	8664		4	0	0	0
	ERH	2079		4	0	0	0
	HIST1H3A	8350		4	0	0	0
	HSPG2	3339		4	0	0	0
	API5	8539		3	0	0	0
	CIAO1	9391		3	0	0	0
	EIF3I	8668		3	0	0	0
	HIST1H2BK	85236		3	0	0	0
	RPS21	6227		3	0	0	0
	SRP14	6727		3	0	0	0
	C1QBP	708		2	0	0	0
	RPL23	9349		2	0	0	0
	SERPINB1	1992		2	0	0	0
	SMARCA5	8467		2	0	0	0
	ACP1	52		1	0	0	0
	ACTR2	10097		1	0	0	0
	ARPC3	10094		1	0	0	0
	CSDE1	7812		1	0	0	0
	DDX6	1656		1	0	0	0
	DUT	1854		1	0	0	0
	EIF2S3	1968		1	0	0	0
	GNB1	2782		1	0	0	0

Name[1]	Symbol[2]	Gene ID[3]	Group[4]	SF1		IgG	
				- RNase	+ RNase	- RNase	+ RNase
	HDLBP	3069		1	0	0	0
	METAP2	10988		1	0	0	0
	MRPL46	26589		1	0	0	0
	PARK7	11315		1	0	0	0
	PDIA4	9601		1	0	0	0
	RPS9	6203		1	0	0	0
	SEC22B	9554		1	0	0	0
	SMC1A	8243		1	0	0	0

[1] Protein name commonly used in the splicing field.

[2] NCBI ENTREZ symbol.

[3] NCBI ENTREZ GeneID.

[4] Operational classification of spliceosomal proteins according to Hegele et al. (49).
